# CXCL4/PF4 is a predictive biomarker of cardiac differentiation potential of human induced pluripotent stem cells

**DOI:** 10.1038/s41598-019-40915-w

**Published:** 2019-03-15

**Authors:** Fumiya Ohashi, Shigeru Miyagawa, Satoshi Yasuda, Takumi Miura, Takuya Kuroda, Masayoshi Itoh, Hideya Kawaji, Emiko Ito, Shohei Yoshida, Atsuhiro Saito, Tadashi Sameshima, Jun Kawai, Yoshiki Sawa, Yoji Sato

**Affiliations:** 1 Division of Cell-Based Therapeutic Products, National Institute of Health Sciences, 3-25-26 Tonomachi, Kawasaki-ku, Kawasaki, Kanagawa 210-9501 Japan; 2 Department of Cardiovascular Surgery, Osaka University Graduate School of Medicine, 2-2 Yamada-oka, Suita, Osaka 565-0871 Japan; 3 Department of Cellular & Gene Therapy Products, Osaka University Graduate School of Pharmaceutical Sciences, 1-6 Yamadaoka, Suita, Osaka 565-0871 Japan; 4 Terumo Corporation, 1500 Inokuchi, Nakai-machi, Ashigarakami-gun, Kanagawa 259-0151 Japan; 5 Preventive Medicine and Diagnosis Innovation Program, RIKEN Center, 1-7-22, Suehirocho, Tsurumi-ku, Yokohama, Kanagawa 230-0045 Japan; 6 Preventive Medicine and Applied Genomics Unit, RIKEN Center for Integrative Medical Sciences, 1-7-22 Suehirocho, Tsurumi-ku, Yokohama, Kanagawa 230-0045 Japan; 7 Department of Quality Assurance Science for Pharmaceuticals, Nagoya City University Graduate School of Pharmaceutical Sciences, 3-1 Tanabe-dori, Mizuho-ku, Nagoya, Aichi 467-8603 Japan; 8 Department of Translational Pharmaceutical Sciences, Graduate School of Pharmaceutical Sciences, Kyushu University, 3-1-1 Maidashi, Higashi-ku, Fukuoka, Fukuoka 812-8582 Japan; 9 LiSE Laboratory, Kanagawa Institute of Industrial Science and Technology, 3-25-13 Tonomachi, Kawasaki-ku, Kawasaki, Kanagawa 210-0821 Japan

## Abstract

Selection of human induced pluripotent stem cell (hiPSC) lines with high cardiac differentiation potential is important for regenerative therapy and drug screening. We aimed to identify biomarkers for predicting cardiac differentiation potential of hiPSC lines by comparing the gene expression profiles of six undifferentiated hiPSC lines with different cardiac differentiation capabilities. We used three platforms of gene expression analysis, namely, cap analysis of gene expression (CAGE), mRNA array, and microRNA array to efficiently screen biomarkers related to cardiac differentiation of hiPSCs. Statistical analysis revealed candidate biomarker genes with significant correlation between the gene expression levels in the undifferentiated hiPSCs and their cardiac differentiation potential. Of the candidate genes, *PF4* was validated as a biomarker expressed in undifferentiated hiPSCs with high potential for cardiac differentiation in 13 additional hiPSC lines. Our observations suggest that *PF4* may be a useful biomarker for selecting hiPSC lines appropriate for the generation of cardiomyocytes.

## Introduction

Human induced pluripotent stem cells (hiPSCs) are capable of differentiating into various tissues^1^, thereby acting as a source of cells for regenerative medicine and drug discovery^2–8^. Technological advancements in the development of disease-specific hiPSCs from somatic cells of patients have enabled the study of the pathology of rare diseases^9,10^. Several studies have suggested that the direction of differentiation of tissues derived from the endoderm, mesoderm, and ectoderm varies depending on the line of human embryonic stem cells (hESCs) and hiPSCs^11–13^. Variation in the direction of differentiation among hiPSC lines is the result of differences in somatic tissue of origin and epigenetic changes^14–16^. As the genetic backgrounds of the somatic cells used to derive hiPSCs differ significantly, the epigenetic variation between hiPSCs and hESCs is large^17^.

Biomarkers are required for selecting suitable hiPSC lines with high differentiation potential for specific tissues. Several studies have previously investigated biomarkers associated with differentiation potential of hiPSCs^18–24^. However, current pluripotency markers such as *OCT-4*, *LIN28*, and *NANOG* cannot be used to distinguish the direction of differentiation.

The purpose of the present study was to identify a biomarker for predicting efficient cardiac differentiation that can be used for selecting individual hiPSC lines by comparing the gene expression profiles of undifferentiated hiPSC lines with varying cardiac differentiation potential. Biomarkers have been searched using single genome-wide analyses^25–27^. However, selection of appropriate genes from among the many candidate genes while minimizing the occurrence of false positives using this approach is challenging. In this study, we hypothesized that biomarkers can be selected using three different platforms of genetic analyses. We comprehensively analysed the gene expression of hiPSCs using cap analysis of gene expression (CAGE), mRNA array, and microRNA array to screen for biomarkers of cardiac differentiation potential. CAGE has been used to analyse transcription start sites and can measure the activity of alternative promoters via absolute quantitation. In contrast, microarray analysis has been used to quantify transcript expression in samples based on the intensity ratio of the hybridisation signal. Our proposed method of using three gene analysis platforms for identifying novel predictive biomarkers of hiPSCs with high cardiac differentiation potential will identify useful genes that can be important for selecting desired hiPSC lines.

## Results

### Outline of the workflow for selecting predictive biomarkers for cardiac differentiation

**Figure 1:**
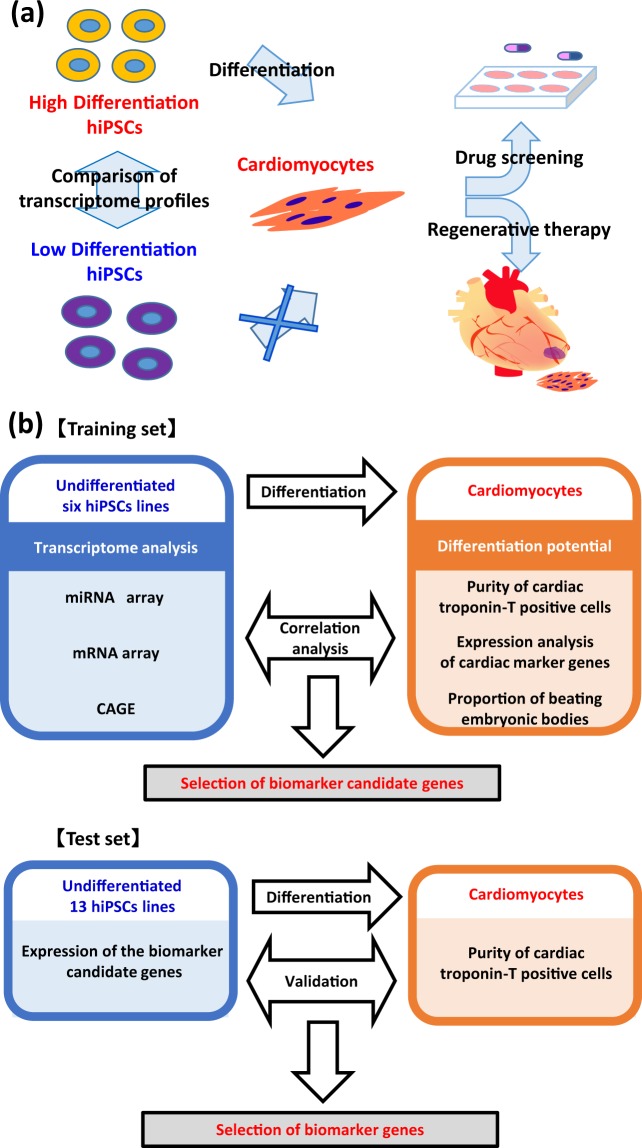
Strategy for identification of biomarkers for cardiac differentiation. (**a**) Schematic illustration of the experimental design. (**b**) Workflow for selecting biomarker candidate genes to predict the cardiac differentiation potential of hiPSCs.

To compare the *in vitro* cardiac differentiation efficiency of hiPSC lines, six hiPSC lines were cultured and differentiated into cardiomyocytes under identical conditions as a training set (Supplementary Table 1). Two types of human somatic tissues were used to establish hiPSCs, namely, dermal fibroblasts and cord blood cells. Five hiPSC lines were generated using retroviral vectors and one hiPSC line using episomal vectors. We performed miRNA array, mRNA array, and CAGE on the undifferentiated hiPSCs to develop comprehensive transcript expression profiles of the undifferentiated hiPSCs. Next, we analysed the cardiomyocytes derived from hiPSCs using flow cytometry, quantitative reverse transcription-polymerase chain reaction (qRT-PCR), immunostaining, and beating analysis, and then determined the cardiac differentiation efficiency ranking. Based on the ranking, the hiPSCs lines were divided into high and low purity groups. To select candidate genes for predictive biomarkers, we compared the mRNA and microRNA (miRNA) expression and the transcription start sites (TSS) in undifferentiated hiPSCs to those of the high and low differentiation groups. Finally, using 13 hiPSC lines as a test set, we examined whether hiPSC lines with high capability of cardiomyogenic differentiation could be selected using the biomarker candidates (Fig. 1).

**Figure 2:**
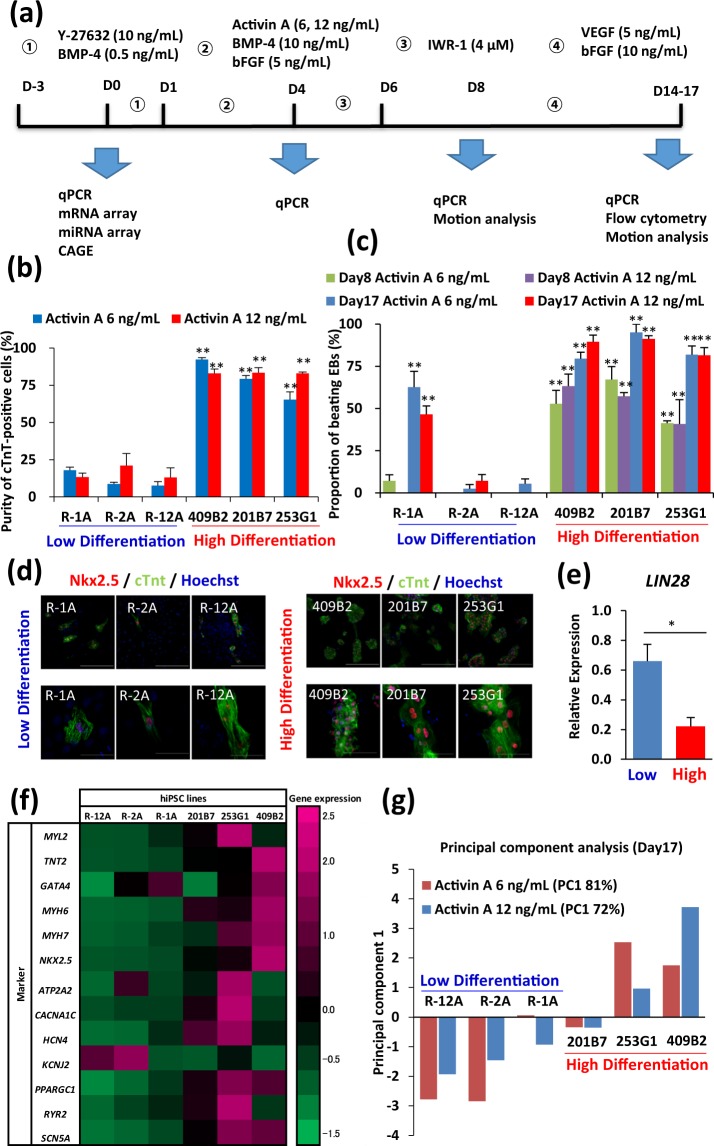
Differences in cardiac differentiation capability among hiPSC lines. (**a**) Schematic of the culturing process for cardiac differentiation in EB suspension cultures. (**b**) Comparison of the cardiac differentiation ability of hiPSC lines using flow cytometric analysis at 17 d post-induction of cardiac differentiation with different concentrations of Activin A; 6 ng/mL and 12 ng/mL. Data are expressed as mean ± SEM (n = 3). **p < 0.01 vs. R-2A, ANOVA and Dunnett’s test. (**c**) Proportion of rhythmic and synchronous beating EBs at 8 d and 17 d post-differentiation with different concentrations of Activin A; 6 ng/mL and 12 ng/mL. Data are expressed as mean ± SEM (n = 3). **p < 0.01 vs. R-2A, ANOVA and Dunnett’s test. (**d**) Immunofluorescence of hiPSC-derived cardiomyocytes for cardiac-specific markers. Micrographs show cTnT (green), Nkx2.5 (red), and Hoechst (blue) staining. Upper panels show low magnification and lower panels high magnification. Scale bars in upper panels = 300 μm, in lower panels = 100 μm. (**e**) Expression of an undifferentiated hiPSC marker gene (*LIN28*) among hiPSC lines at 17 d post-cardiac differentiation. Data are expressed as mean ± SEM (n = 6). *p < 0.05, *t*-test. All mRNA values are shown as fold change relative to the expression of R-2A in Low differentiation group. (**f**) Heat map of cardiomyocyte-related genes and maturation-related genes among six hiPSC lines. (**g**) Principal component analysis of cardiac differentiation ability among six hiPSC lines at 17 d post-cardiac differentiation with different concentrations of Activin A; 6 ng/mL and 12 ng/mL (FC1, first principal component scores).

For cardiac differentiation of the hiPSCs, we used an embryoid body (EB) differentiation method (Fig. 2a) based on a previous protocol with some modifications^28–30^, as EBs formed using three-dimensional (3D) differentiation methods can be easily scaled up for clinical application of hiPSC-derived cardiomyocytes. As the formation of EBs is a critical process in cardiac differentiation, several methods of inducing EB formation from hiPSCs have been established to promote efficient differentiation^31^. Therefore, we compared the cardiac differentiation efficiency at the EB formation step of the cardiac differentiation process between two methods, the small cell clump method and the single cell method. The small cell clump method enzymatically disrupted hiPSC colonies into small cell clumps, whereas the single cell method enzymatically dissociated them into single cells. After evaluating cardiomyocytes derived from 253G1 hiPSCs for cardiac troponin T (cTnT) expression using flow cytometry, we concluded that EB formation using the single cell method was more efficient and robust with respect to cardiomyocyte differentiation than the small cell-clump method (single cells, 83.4 ± 1.2%, n = 110 vs. small clumps, 37.2 ± 2.2%, n = 46) (Supplementary Fig. 1). In subsequent experiments, we determined the protocol for the use of the single cell method for cardiac differentiation as shown in Fig. 2a.

### Differences in cardiac differentiation abilities of hiPSC lines

First, we analysed the expression of undifferentiated cell markers of each hiPSC line using qRT-PCR. In addition to the similarities in cell morphology with pluripotent stem cells, the expression levels of the undifferentiated cell markers showed no significant difference among the six hiPSC lines (Supplementary Fig. 2). Next, we subjected all the hiPSC lines to cardiac differentiation. A previous study reported that the highest number of cardiomyocytes was obtained from the mesoderm induced with 10 ng/mL BMP-4 and 6 ng/mL of Activin A, while higher or lower levels of Activin A and BMP-4 showed lower cardiomyocyte differentiation efficiencies^32^. Based on these findings, we applied two different concentrations of Activin A (6 ng/mL and 12 ng/mL) to the hiPSC lines with 10 ng/mL BMP-4, as shown in the Supplementary Information. Flow cytometry analysis revealed differences in the prevalence of cTnT-positive cells among hiPSC lines. The percentage of cTnT-positive cells ranged from 7.7 ± 2.6% in R-12A cells to 92.3 ± 1.2% in 409B2 cells (Fig. 2b).

We calculated the proportion of beating EBs in differentiated hiPSCs at 8 d and 17 d post-differentiation. In the high differentiation group, approximately 50% and 90% of the EBs demonstrated rhythmical and synchronous beating at 8 d and 17 d post-differentiation, respectively. In contrast, the proportion of beating EBs was low in the low differentiation group, even after 17 d of differentiation (Fig. 2c). With respect to cardiac differentiation capability, hiPSC lines could be categorised into two distinct groups, the low (R-1A, R-2A, and R-12A) and high differentiation groups (409B2, 201B7, and 253G1). Similarly, cardiomyocytes derived from the hiPSC lines in the high differentiation group expressed *TNT2* and *NKX2.5* at >20-fold higher levels than those in the low differentiation group (Supplementary Fig. 3). Differences in the concentrations of Activin A did not affect the percentage of cTnT-positive cells in the hiPSC lines with low differentiation efficiency. Immunostaining also revealed that the number of cTnT-positive and NKX2.5-positive cells in the cardiac population was higher in the high differentiation group than in the low differentiation group (Fig. 2d). Furthermore, in the low differentiation group, we detected many α-SMA and vimentin-positive smooth muscle cells and fibroblasts (Supplementary Fig. 4), which was consistent with the results of previous reports^33,34^.

The presence of residual undifferentiated cells after differentiation is a critical issue regarding clinical application of hiPSC-derived cardiomyocytes^4,35–38^. Therefore, we compared the expression of an hiPSC marker gene (*LIN28*) between the high and low differentiation groups on day 17 after the induction of differentiation. The expression of the undifferentiated hiPSC marker *LIN28*^39^ was significantly higher in the low differentiation group than in the high differentiation group, suggesting that the lower efficiency in cardiac differentiation correlated inversely with a higher proportion of residual undifferentiated hiPSCs (Fig. 2e). This underscored the importance of selecting hiPSC lines with high differentiation potential for clinical application of hiPSC-derived cardiomyocytes.

To examine the expression of cardiomyocyte-related genes in EBs, we extracted RNA from hiPSC-derived cardiomyocytes on day 17 after the induction of differentiation and performed qRT-PCR analysis. The expression of cardiomyocyte-related genes in EBs formed after induction with 12 ng/mL Activin A was higher in the high differentiation group than in the low differentiation group. Heat maps demonstrated the relative expression levels of cardiomyocyte-related genes and cardiac maturation-related genes in EBs derived from the hiPSC lines (Fig. 2f). The cardiomyogenic ranking in EBs at 17 d post-differentiation was calculated based on the expression levels of six cardiomyocyte-related genes that were used as markers. We performed principal component analysis (PCA) to determine the rank of cardiac differentiation capacity among the six hiPSC lines. The ranking of hiPSC lines according to their cardiac differentiation potential (from highest to lowest) was 409B2, 253G1, 201B7, R-1A, R-2A, and R-12A (Fig. 2g). The expression of cardiomyocyte-related genes was also high in the high differentiation group of EBs at 9 d post-differentiation. In addition, PCA based on the expression of cardiomyocyte-related genes in EBs demonstrated that the ranking order on day 9 post-differentiation was identical to that on day 17 (Supplementary Fig. 5). We recalculated cTnT-positive rates of EBs at day 17 in the high and low differentiation groups and observed a significant difference in the cTnt-positive rate between the two groups; low = 13.6% ± 1.9 vs. high = 81.1% ± 3.3% (*p* < 0.01) (Supplementary Fig. 6).

### Expression of germ layer-related genes in EBs derived from hiPSC lines

**Figure 3:**
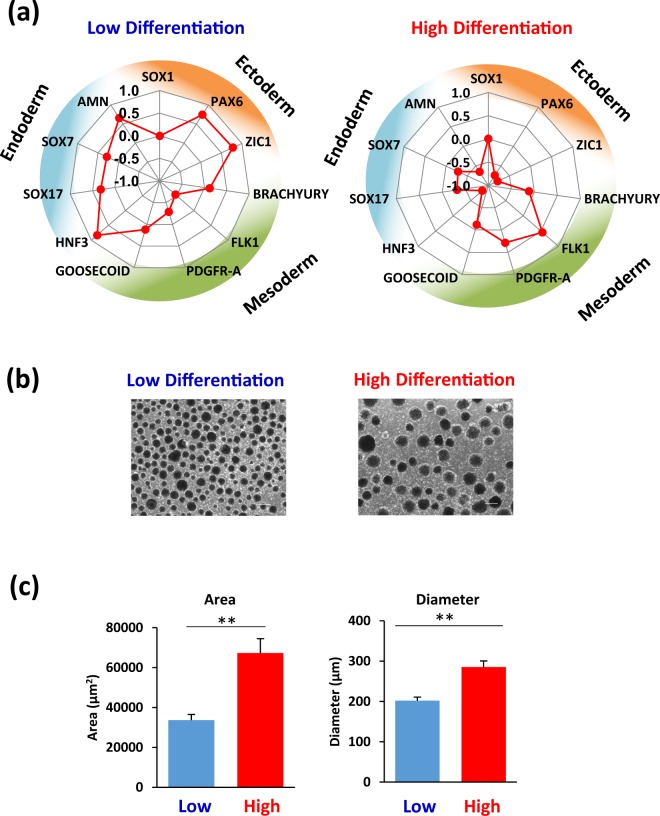
Comparison of differentiation direction of hiPSC lines at 4 d post-cardiac differentiation. (**a**) Radar chart based on expression levels of endoderm, ectoderm, and mesoderm-related genes at 4 d post-cardiac differentiation. (**b**) Images of EBs 6 d post-cardiac differentiation of the training set of hiPSC lines. Scale bars = 300 μm. (**c**) Cross-sectional area and diameter of EBs at 6 d post-cardiac differentiation. Data are expressed as mean ± SEM (n = 6). **p < 0.01, *t*-test.

To determine the differentiation direction of hiPSC lines in the early stage of differentiation, we analysed gene expression of the three embryonic germ layers in EBs on day 4 of cardiac differentiation using qRT-PCR. The radar charts were based on the expression of genes specific to the mesendoderm and mesoderm (*GOOSECOID*, *PDGFR-A*, *FLK1*, and *BRACHYURY*), endoderm (*HNF3*, *SOX7*, *SOX17*, and *AMN*), and ectoderm (*ZIC1*, *SOX1*, and *PAX6*), which enabled assessment of the differentiation direction of the hiPSC lines with high and low differentiation potential. The hiPSC lines in the high differentiation group showed high expression of the mesodermal gene *FLK1* during the early stage of differentiation, whereas the hiPSC lines in the low differentiation group showed high expression of the endodermal gene *AMN* and the ectodermal gene *PAX6* (Fig. 3a). We compared the ranking order of the hiPSC lines for cardiac differentiation potential at 17 d post-differentiation (Fig. 2g) and the expression of germ-layer related genes at 4 d using Spearman’s rank correlation analysis. The expression levels of *FLK1* correlated positively with the cardiac differentiation potential of hiPSC lines (Spearman correlation coefficient (rs) = 0.75, *p* = 0.05). In contrast, the expression of *ZIC1* and *AMN* showed negative correlation (*ZIC1*, rs = −0.57, *p* = 0.18; *AMN*, rs = −0.89, *p* < 0.05) (Supplementary Table 2). Considering that cardiomyocytes are generated from the mesoderm^40^, we inferred that mesodermal genes were upregulated in hiPSCs with high cardiac differentiation potential during the early stage of differentiation. The expression of germ layer-related genes at 4 d post-differentiation reflected a lineage-specific direction of hiPSC lines, suggesting that the differentiation process of EBs can be confirmed by measuring the expression levels of *FLK1*, *ZIC1*, and *AMN*. In addition, we observed that the diameter and cross-sectional area of EBs at 6 d post-differentiation were significantly different between the high and low differentiation groups of hiPSC lines. The mean diameters of the high and low differentiation groups were 286 ± 15 µm and 202 ± 9 µm, respectively (*p* < 0.01), whereas the mean cross-sectional areas of the high and low differentiation groups were 67329 ± 7145 µm^2^ and 33676 ± 2892 µm^2^ (*p* < 0.01), respectively (Fig. 3b,c). During the time course of cardiac differentiation, the diameter of EBs in the high differentiation group was significantly larger than that in the low differentiation group (Supplementary Fig. 7a). We also demonstrated that the number of cells per EB was significantly different between the high and low differentiation groups (Supplementary Fig. 7b). Moreover, the immunostaining image of the section from EBs revealed that the hollow core of the EBs in the high differentiation group was larger than that in the low differentiation group (Supplementary Fig. 7c). Previous studies also reported that the size of EBs was associated with cardiac differentiation in ES cells^31,41^. Therefore, the differences in cell proliferation and the hollow core of EBs may have a significant impact on cell differentiation in EBs, suggesting that the non-invasive measurement of EB size could be useful for process control.

### Comprehensive gene expression analysis of undifferentiated hiPSCs using miRNA and mRNA arrays

**Figure 4:**
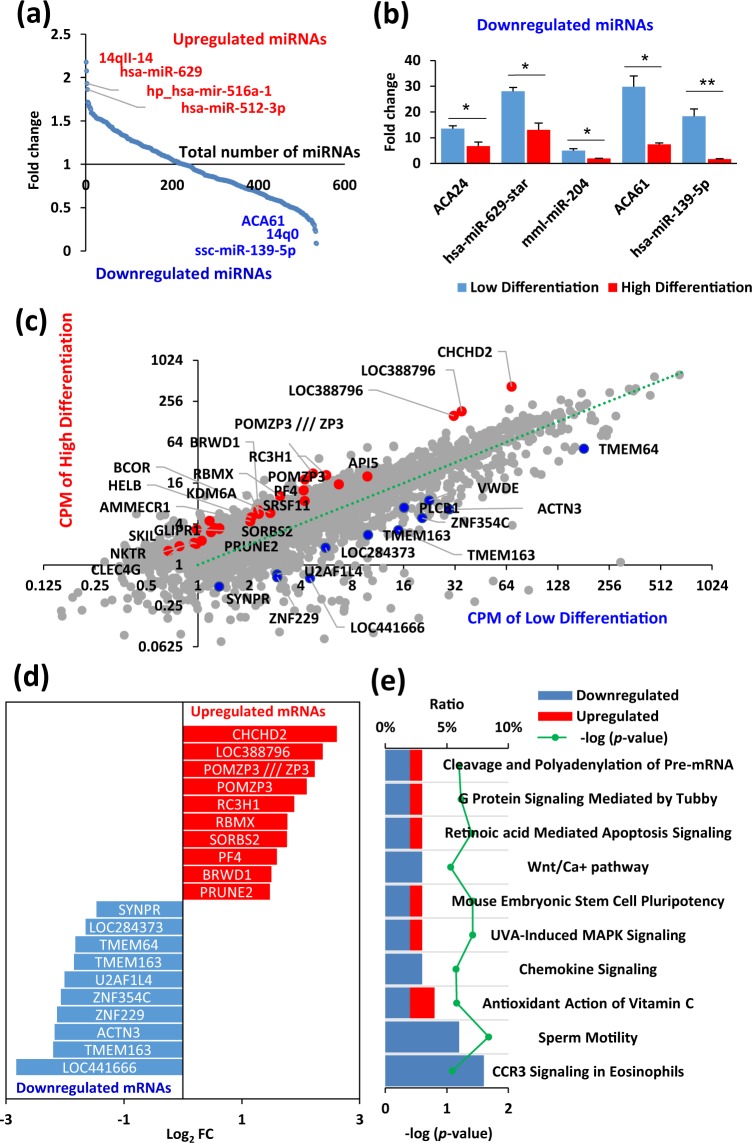
Transcriptome analysis of undifferentiated hiPSCs using miRNA array and GeneChip array. (**a**) Plot showing expression of relative fold change expression between high and low differentiation groups. The x-axis indicates miRNA ranks for relative fold change and the y-axis shows the expression ratio (high/low) based on the differential profiles of 535 miRNAs in hiPSCs. (**b**) The differential expression of miRNAs in the high differentiation and low differentiation groups at a *p* < 0.05 and FC > 2. Data are expressed as mean ± SEM (n = 3). **p < 0.01; *p < 0.05, *t*-test. (**c**) Scatter plot showing counts per million (CPM) of high differentiation (y-axis) vs. CPM of low differentiation (x-axis) from the mRNA array analysis. Red and blue coloured points and gene names indicate mRNAs that were significantly changed (FC > 2 and *p* < 0.01, *t*-test). (**d**) Graph showing the fold changes of the top and bottom differentially expressed genes from a GeneChip analysis comparing the high differentiation group with the low differentiation group. Selected genes have been colour-coded and labelled. Red, top 10 expression in the high differentiation group (*p* < 0.01, *t*-test); blue, top 10 expression in the low differentiation group (*p* < 0.01). (**e**) Pathway analysis of the collective expression levels of interacting genes involved in specific pathways.

Short non-coding RNAs, such as miRNAs, play important roles in silencing targeted genes by regulating post-transcriptional events^42^. Over 500 human miRNAs and small nucleolar RNAs (snoRNAs) have been described and each can regulate hundreds of different mRNAs^43^. Previous reports showed that miRNAs can control cell lineage determination and maturation of hiPSCs, possibly by regulating the transcriptome of undifferentiated hiPSCs^44–46^. Therefore, we performed miRNA array analysis and investigated miRNA expression profiles in the hiPSC lines (Fig. 4a). Differential analysis based on miRNA array comparing the high and low differentiation groups identified three miRNAs and two snoRNAs that were statistically different (*p* < 0.05, FC > 2; Fig. 4b and Supplementary Table 3). The miRNAs were expressed at significantly higher levels in the low differentiation group than in the high differentiation group, suggesting that these miRNAs may be involved in inhibiting the cardiac differentiation of hiPSCs and in maintaining cells in a state of self-renewal. To characterise the miRNA-mediated regulation of cardiac differentiation, miRNA target prediction was performed using the Ingenuity software (Qiagen). In total, 1924 genes were identified as being regulated by has-miR-139, mml-miR-204, and hsa-miR-629 (Supplementary Dataset 1).

Next, gene expression of the high and low differentiation groups was compared quantitatively to identify genes that were expressed in undifferentiated hiPSCs and may regulate cardiac differentiation. To perform a comprehensive analysis of the gene expression of undifferentiated hiPSCs, the mRNA array was used to identify differentially expressed transcripts in the high differentiation group compared to that in the low differentiation group. We identified 20 upregulated genes and 11 downregulated genes related to cardiac differentiation capability (FC > 2 and *p* < 0.01; Fig. 4c,d). Ingenuity canonical pathway analysis showed that the genes differentially expressed between the high and low differentiation groups were involved in chemokine signaling and the Wnt/Ca+ signaling pathway (Fig. 4e).

### Profiling of TSS expression in undifferentiated hiPSCs using CAGE

**Figure 5:**
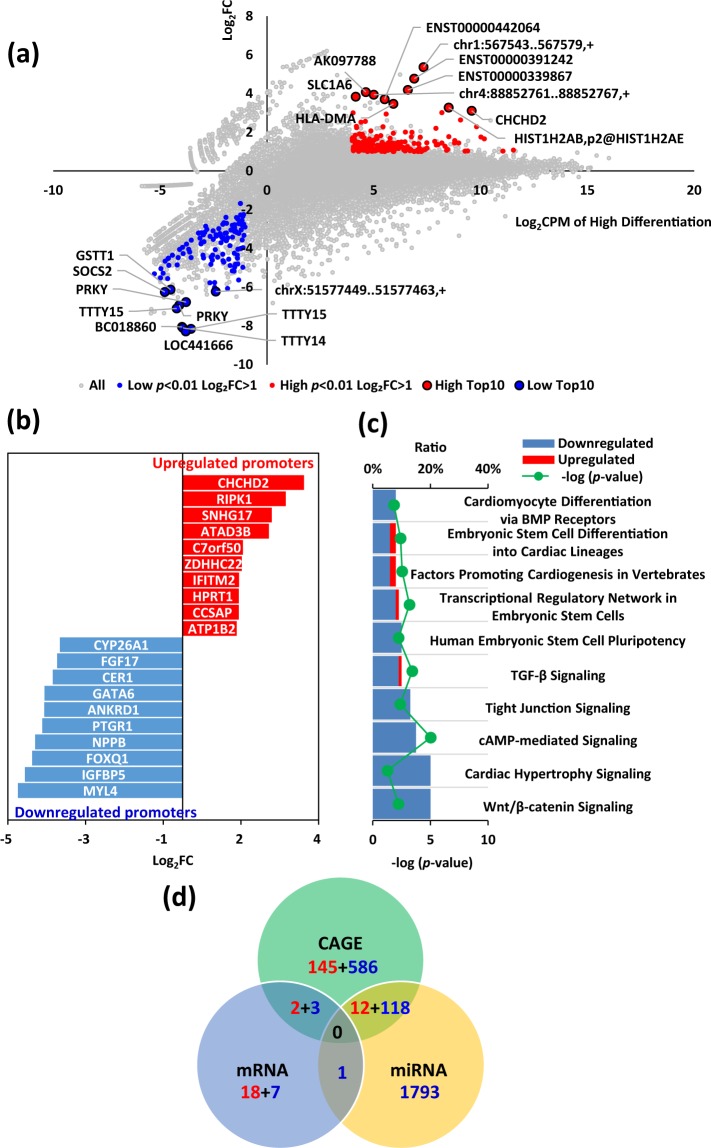
Undifferentiated hiPSC transcriptome profiling using CAGE. (**a**) Scatter plot shows fold changes of individual genes from CAGE. (**b**) Graph showing the fold changes of the top and bottom differentially expressed promoters according to CAGE comparing the high differentiation group with the low differentiation group. Selected genes have been color-coded and labelled. Red, top 10 expression in the high differentiation group; blue, top 10 expression in the low differentiation group (FDR (false discovery rate) < 0.01, FC > 2). (**c**) Pathway analysis of the collective expression levels of interacting genes involved in specific pathways. (**d**) List of predictive biomarkers for cardiac differentiation. Venn diagram analysis visualising the overlap among the candidate genes identified using CAGE, mRNA array, and miRNA array analyses. The number of upregulated and downregulated mRNAs in hiPSC lines of the high differentiation group compared to that in the low differentiation group is indicated by red (up) and blue (down) colours, respectively.

To further investigate the correlation between the genetic state of hiPSC lines and cardiac differentiation potential, we comprehensively compared TSS expression between the high and low differentiation groups using CAGE^47–49^, which allowed identification of specific promoters for hiPSC differentiation. We identified 159 upregulated-transcripts and 707 downregulated-transcripts with more than twofold change in expression and FDR (false discovery rate) less than 0.01 in the high differentiation group compared to that in the low differentiation group (Fig. 5a). The genes identified using CAGE with the highest differentially expressed fold-changes in either direction are shown in Fig. 5b. Pathway analysis of these differentially expressed genes demonstrated that genes related to BMP receptors and human embryonic stem cell pluripotency were possibly involved in cardiac differentiation (Fig. 5c). In addition, Venn diagram analysis indicated that five genes (*CHCHD2*, *PF4*, *ZNF229*, *ZNF354C*, and *LOC441666*) were differentially expressed in both mRNA array and CAGE, 130 genes were differentially expressed in CAGE and were targets of differentially expressed miRNAs, and one gene (*TMEM64*) that was differentially expressed in the mRNA array was the target of differentially expressed miRNAs. Genes were categorised as positive predictors (upregulated in the high differentiation group) and negative predictors (downregulated in the high differentiation group) of cardiac differentiation potential (Fig. 5d and Supplementary Dataset 1). In addition to these eight genes (*CHCHD2*, *PF4*, *ZNF229*, *TMEM64*, *FGF17*, *GATA6*, *ANKRD1*, and *IGFBP5*) identified using multiple analysis platforms, we selected 14 genes related to cell differentiation that were differentially expressed in at least one platform. In total, we selected 22 genes as biomarker candidates and listed their cellular roles (Tables 1, 2).

**Table 1:** Positive predictive biomarker candidates for cardiac differentiation of hiPSC lines.

Gene name	Analysis	Entrez gene name	Role in cell
*CHCHD2*	CAGE & mRNA	coiled-coil-helix-coiled-coil-helix domain containing 2	phosphorylation in, differentiation, expression in, migration by, signaling in, formation in, formation
*PF4*	CAGE & mRNA	platelet factor 4	proliferation, chemotaxis, activation, binding, growth, chemoattraction, differentiation, migration, survival, adhesion
*KDM6A*	mRNA	lysine demethylase 6A	differentiation, identity, expression in, remodelling, cell viability, proliferation
*BCOR*	mRNA	BCL6 corepressor	differentiation, formation
*POMZP3*	mRNA	POM121 and ZP3 fusion	formation
*RBMX*	mRNA	RNA binding motif protein, X-linked	alternative splicing by, homologous recombination in, expression in
*RC3H1*	mRNA	ring finger and CCCH-type domains 1	proliferation, homeostasis, quantity, number, expression in, abnormal morphology, degradation in, differentiation
*GLIPR1*	mRNA	GLI pathogenesis related 1	apoptosis, sensitivity, destruction in, cell cycle progression, transactivation in, degradation in, binding in, ubiquitination in
*RIPK1*	CAGE	receptor interacting serine/threonine kinase 1	apoptosis, activation in, cell death, necroptosis, necrosis, expression in, production in, survival, proliferation, formation in
*C7orf50*	CAGE	chromosome 7 open reading frame 50	unknown

**Table 2:** Negative predictive biomarker candidates for cardiac differentiation of hiPSC lines.

Gene name	Analysis	Entrez gene name	Role in cell
*ZNF229*	CAGE & mRNA	zinc finger protein 229	unknown
*PLCB1*	mRNA	phospholipase C beta 1	differentiation, expression in, activation in, cell death, G2/M phase transition, loss, binding, size, hypertrophy, fusion
*TMEM64*	mRNA & miRNA	transmembrane protein 64	differentiation
*PTGR1*	CAGE	prostaglandin reductase 1	survival
*FOXQ1*	CAGE	forkhead box Q1	formation by, expression in, quantity, migration, proliferation
*MYL4*	CAGE	myosin light chain 4	unknown
*FGF17*	CAGE & miRNA	fibroblast growth factor 17	proliferation, abnormal morphology, phosphorylation in, survival
*GATA6*	CAGE & miRNA	GATA binding protein 6	differentiation, expression in, proliferation, apoptosis, transcription in, transactivation in, specification, growth, abnormal morphology
*ANKRD1*	CAGE & miRNA	ankyrin repeat domain 1	apoptosis, response, differentiation, cell viability, expression in, colony formation
*IGFBP5*	CAGE & miRNA	insulin like growth factor binding protein 5	growth, migration, apoptosis, proliferation, survival, translation in, differentiation, cell spreading, expression in, quantity
*WNT3*	CAGE	Wnt family member 3	expression in, binding in, accumulation in, signaling in, transcription in, phosphorylation in, proliferation, differentiation, stabilization in
*IGF2*	CAGE	insulin like growth factor 2	proliferation, differentiation, growth, migration, phosphorylation in, apoptosis, expression in, activation in, survival, quantity

### Validation of 22 candidate genes as predictors of cardiac differentiation potential in a test set of 13 hiPSC lines

**Figure 6:**
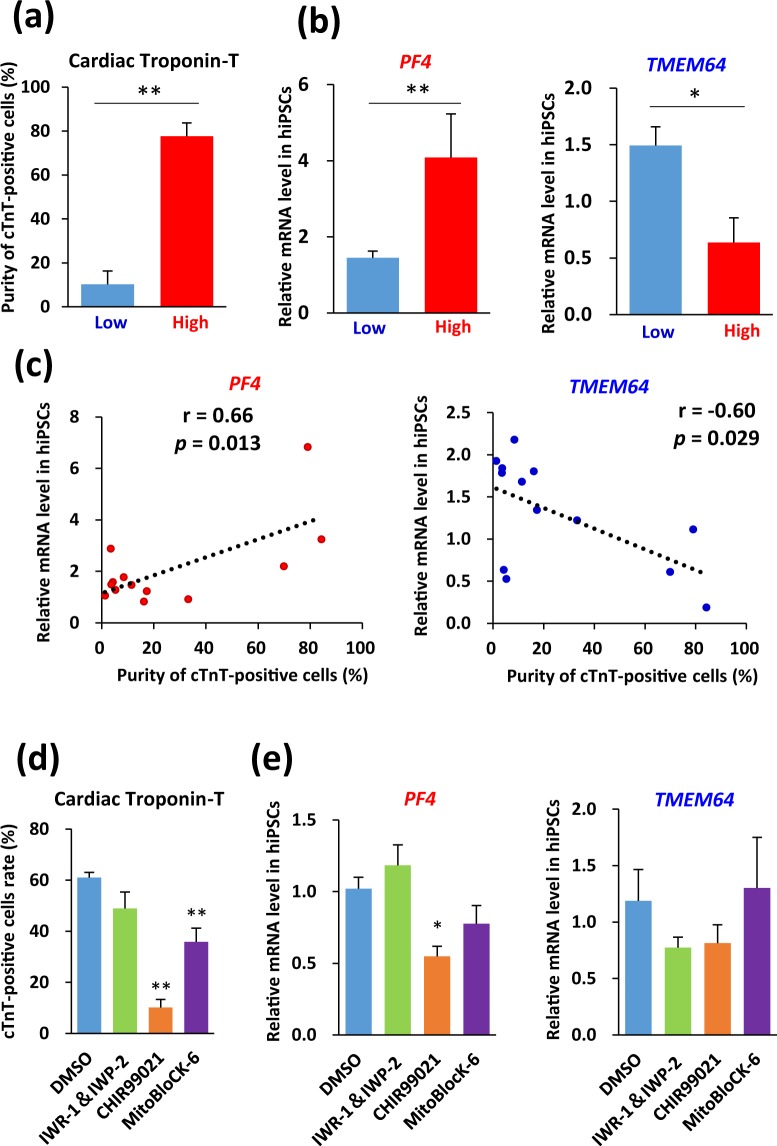
Validation results for expression of 22 genes in a test set of 13 hiPSC lines. (**a**) Percentage of cTnT-positive cells generated from hiPSCs in high and low differentiation groups at 17 d post-differentiation in a test set of hiPSC lines. Data are expressed as mean ± SEM (n = 3–10). **p < 0.01, *t*-test. (**b**) *PF4* and *TMEM64* mRNA expression levels in the high and low differentiation groups were quantified using qRT-PCR. Data are expressed as mean ± SEM (n = 3–10). **p < 0.01; *p < 0.05, *t*-test. (**c**) *PF4* and *TMEM64* mRNA levels in undifferentiated hiPSCs correlated with their cardiac differentiation efficiency along with r and *p* values. All mRNA values are shown as fold change relative to the expression of PCi-1533. Each dot indicates the expression level in each hiPSC line. (**d**) Percentage of cTnT-positive cells generated from hiPSCs with a common genetic background (253G1) incubated with IWR-1 & IWP-2, CHIR99021, and Mitoblock-6 at 17 d post-differentiation. Data are expressed as mean ± SEM (n = 6). **p < 0.01vs. DMSO, ANOVA and Dunnett’s test. (**e**) *PF4* and *TMEM64* expression levels of the hiPSCs incubated with IWR-1 & IWP-2, CHIR99021, and Mitoblock-6. All mRNA values are shown as fold change relative to the expression of mRNA in DMSO-treated control cells. Data are expressed as mean ± SEM (n = 6). *p < 0.05 vs. DMSO, ANOVA and Dunnett’s test.

Differential gene expression between the high and low differentiation groups of undifferentiated hiPSCs revealed potential candidate genes related to cardiac differentiation potential. The expression of 22 biomarker candidate genes that predicted the cardiac differentiation capacity of hiPSC lines was confirmed in 13 additional hiPSC lines. A previous report showed that culture conditions affect the pluripotency and cardiac differentiation of hiPSCs^50^. Therefore, hiPSC lines of the test set were evaluated under on-feeder and feeder-free conditions to determine the effects of extracellular matrix on cardiac differentiation (Supplementary Table 4). To validate the biomarker candidate genes using a method other than CAGE, and mRNA and miRNA arrays, we measured the expression of each biomarker candidate gene in these undifferentiated hiPSC lines using qRT-PCR. The cells were then differentiated into cardiomyocytes using the protocol shown in Fig. 2a. The differentiated cells of each hiPSC line were divided into high and low differentiation groups based on whether the cTnT-positive rate was 50% or higher, and the cTnt-positive rates were recalculated for the differentiated hiPSC lines of each group (Fig. 6a). Next, we compared the expression levels of the biomarker candidate genes between the two groups. Twenty of the 22 genes exhibited no significant difference in expression between the two groups (Supplementary Fig. 8). These observations suggested that those 20 candidate genes were false positives. Two of the candidate genes in the test set correlated either positively or negatively with cardiac differentiation potential (Fig. 6b). The gene that positively correlated with cardiac differentiation potential was *PF4* (Fig. 6c, left), which is known to be involved in cellular functions, including proliferation, chemotaxis, activation, binding, growth, chemoattraction, differentiation, migration, survival, and adhesion. PF4 has been reported as one of the most potent antiangiogenic chemokines influencing angiogenesis^51^, suggesting that *PF4*-high expressing hiPSC lines may be efficiently induced to differentiate into cardiomyocytes. In addition, *TMEM64* was found to negatively correlate with cardiac differentiation potential (Fig. 6c, right). Reports show that *TMEM64* is involved in WNT signaling^52^, possibly affecting the differentiation of cells into cardiomyocytes. Although WNT3^20^, IGF2^18^, and CHCHD2^19^ have been reported as differentiation markers, results from the present study revealed only low correlation of these genes as a predictive marker for cardiac differentiation.

As WNT signaling and mitochondrial function have been reported to play critical roles in the cardiac differentiation of hiPSCs^20,53^, we attempted to differentiate hiPSCs after treatment with WNT signaling inhibitors IWR-1 and IWP-2, a WNT signaling activator CHIR99021, and a mitochondrial function inhibitor MitoBlock-6^54^. We observed that CHIR99021 and Mitoblock-6 significantly decreased the cardiac differentiation efficiency compared to that of vehicle (Fig. 6d). In addition, treatment with CHIR99021 suppressed *PF4* expression in hiPSC lines of the high differentiation group, indicating that the activity of WNT signaling was associated not only with the cardiomyocyte differentiation efficiency, but also with *PF4* expression. Furthermore, the 2D plots of the raw data points of Fig. 6 indicated a significant correlation between *PF4* gene expression in hiPSCs with a common genetic background and the purity of cTnT-positive cells after their differentiation (Supplemental Fig. 9a). In contrast, *TMEM64* expression did not correlate with changes in cardiac differentiation potential (Fig. 6e and Supplemental Fig. 9b).

Finally, we performed experiments to examine the functional significance of PF4 in cardiomyocyte differentiation of hiPSCs. In brief, cardiomyocyte differentiation of hiPSCs, which were cultured in the presence of PF4 (1 μM)^55^ for 2 days prior to differentiation induction, led to 1.3- to 10.0-fold higher expression levels of cardiomyocyte-specific genes (*MYH7*, *MYL2* and *TNT2*) at 14 d post-differentiation, compared with the controls (Supplementary Fig. 10). Similarly, PF4 treatment of hiPSCs resulted in an increased number of strong beating EBs, compared to the controls (Supplementary Video 1). The cross-sectional area of the EBs, which was associated with the cardiomyogenic potential of hiPSC lines (Fig. 3c), was significantly larger in the PF4-treated group, compared with that in the control group. In addition, the proportion of beating EBs in the PF4-treated group was significantly higher than that in the control group (Supplementary Fig. 11).

## Discussion

Transcriptome comparison of hiPSC lines between the high and low differentiation groups identified *PF4* as a novel biomarker that may be used to distinguish between high and low cardiac differentiation potential of hiPSC lines. For clinical application of hiPSC-derived cardiomyocytes, suitable hiPSC lines must be selected, from which highly purified cardiomyocytes containing minimal undifferentiated hiPSCs can be generated. Our results suggest that the cardiac differentiation potential of individual hiPSC lines can be predicted by assessing *PF4* expression in hiPSCs. In addition, it enables to reduce the potential risk of tumorigenicity from residual undifferentiated hiPSCs in the end product. In our study, we identified *PF4* as a marker for selection of hiPSCs as a raw material for cardiomyocyte production. The data from the test set of 13 hiPSC lines suggest the robustness of this biomarker, though the usefulness of *PF4* as a biomarker could be changed by further optimization of the differentiation protocol.

We showed variability in cardiac differentiation potential of hiPSCs lines by assessing cardiomyocytes differentiated from hiPSCs. We focused on the gene expression profiles of hiPSC lines to identify predictive biomarkers for hiPSCs with cardiac differentiation potential. As factors specific to the original cells and reprogramming methods are both known to influence the differentiation efficiency of hiPSC lines^56^, we used hiPSCs that were derived from origins such as dermal fibroblasts, umbilical cord blood, and other somatic cells in our analyses. We also used hiPSCs reprogrammed with retroviral and episomal vectors. Our results showed that hiPSC lines derived from skin fibroblasts tended to differentiate more efficiently into cardiomyocyte than hiPSC lines derived from umbilical cord blood. Furthermore, we used 253G1, 201B7, and 409B2, which were generated from the same individual and dermal fibroblasts, and different reprogramming methods as shown in Supplementary Table 1. Our results demonstrate that the hiPSC lines expressing high levels of PF4 had a higher capacity to differentiate into cardiomyocytes. The levels of PF4 and the purity of cardiomyocytes were not different among the three hiPSC lines (data not shown). Taken together, these findings suggest that their efficiencies for cardiomyocyte differentiation do not depend on the reprogramming methods. Considering the epigenetic influence of somatic cells on differentiation, analysis of DNA methylation profiles of hiPSC lines and large-scale differentiation experiments might be necessary in the future, as has been reported previously^13,14^.

In the present study, we evaluated hiPSCs in an undifferentiated state using three comprehensive gene expression analysis approaches that included miRNA array, mRNA array, and CAGE, as multiple strategies for identifying biomarkers that are clearly required to reduce the number of false positives. In the process of identifying biomarkers for differentiation potential, the three platforms for quantitating transcript expression allowed us to better understand the mechanisms underlying hiPSC differentiation into cardiogenic lineages. Furthermore, the three independent genetic analyses identified common genes by comparing the transcript expression patterns between hiPSC lines with high and low differentiation potential. Validation using qRT-PCR analysis of candidate genes from hiPSC lines of the test set further decreased the number of false positives. Indeed, *PF4* expression overlapped as a candidate gene between the CAGE and GeneChip analyses, and 20 false positive markers were eliminated using the qRT-PCR-based validation. Using our comprehensive gene analysis data of commercially available undifferentiated hiPSCs, researchers can search for new differentiation biomarkers in desired cell types such as blood, liver, and neural cells.

Moreover, the marker for cardiac progenitors was used to identify the cardiomyogenic potential of the hiPSC lines at the early stage of differentiation^32,57^. However, our results demonstrated that the cardiac differentiation capacity could be predicted by measuring the expression of *PF4* in the undifferentiated hiPSCs. PF4, a known heparin neutralising factor released from platelets, plays a key role in the activation and differentiation of monocytes and macrophages and is associated with systemic sclerosis and cancer^58^. In addition, PF4 levels in the blood have been proposed as biomarkers for determining cancer types^59^. We demonstrated that *PF4* expression was decreased in hiPSC lines with low cardiac differentiation potential and that higher percentage of residual undifferentiated cells remained after differentiation in the low differentiation group of hiPSC lines than in the high differentiation group. Taken together, these results suggested that *PF4* can be used to distinguish among hiPSC lines associated with tumorigenicity after induction of differentiation. The pharmacological experiment using modulators of WNT signaling and mitochondrial function also showed that *PF4* mRNA level in hiPSCs with a common genetic background has a positive association/correlation with the cardiac differentiation capacity. In addition, we found that the pretreatment of hiPSCs with PF4 enhanced the cardiac differentiation potential of hiPSCs, suggesting that PF4 has a cause-and-effect relationship with their cardiac differentiation. These observations suggest that *PF4* gene expression, which could vary not only between hiPSC lines but also between pharmacological conditions, reflect the potential of hiPSCs to differentiate into cardiomyocytes and can thus be used as a quality control marker of hiPSCs.

Although PF4 was found to facilitate cardiac differentiation of hiPSCs, the mechanism underlying its regulation of cardiac differentiation remains unknown. As PF4 is a chemokine that suppresses FGF2-dependent ERK phosphorylation^60^, we presumed that it may possibly modulate FGF signals in hiPSCs. FGF2 and BMP signals are important for inducing differentiation of cells into cardiomyocytes^61,62^, and FGF2/ERK signals suppress BMP signaling-induced Smad1 phosphorylation in ES cells. Indeed, the expression of pluripotency markers and proliferative ability are not altered in FGF knockout stem cells, which rather show difficulty in differentiating into neural cells^63,64^. Therefore, suppression of the FGF2 signal via PF4 may play a key role in directing cardiac differentiation in hiPSCs.

In addition, we observed that WNT activation reduced *PF4* expression in hiPSCs, which implies a role of WNT signaling in the regulation of cardiac differentiation via *PF4*. These results suggest that *PF4* is a novel biomarker for selecting hiPSC lines that are likely to differentiate into cardiomyocytes, which can be used in regenerative therapy and drug screening. In the present study, we focused on the differentiation process of hiPSC lines. However, the gene expression patterns of hESC lines and the mechanism for the maintenance of their pluripotency are known to be similar to those of hiPSC lines. Thus, it seems likely that our biomarker could be applicable to hESC lines.

In summary, hiPSCs with high cardiomyogenic potential showed high expression levels of *PF4*, suggesting that this gene may be used as a biomarker for the selection of hiPSCs that are suitable for generating cardiomyocytes. In the current study, we demonstrated the feasibility of using our approach to efficiently select critical biomarkers in hiPSC lines, which can then be used for screening the cardiac differentiation potential of hiPSCs. In addition to the identification of *PF4*, this new approach will facilitate the identification of novel biomarkers for the differentiation potential of hiPSCs. This new strategy may be beneficial in selecting novel biomarkers and in eliminating false-positives.

## Materials and Methods

### Cell culture and differentiation of hiPSCs

We used commercially available hiPSC lines as listed in Supplementary Tables 1, 4. To differentiate hiPSCs into cardiomyocytes, we modified a previously described protocol^28–30^, the details of which are provided in the Supplemental Experimental procedures (Cell culture and differentiation of hiPSCs).

### Affymetrix miRNA labelling, array hybridisation, and data pre-processing

Undifferentiated hiPSCs were maintained on Matrigel (Corning, New York, NY, USA) -coated dishes in mTeSR1 medium (StemCell Technologies, Vancouver, Canada). Total RNA was isolated from hiPSC lines using a miRNeasy mini kit (Qiagen, Hilden, Germany) and treated with DNase I according to the manufacturer’s instructions. Total RNA containing low molecular weight RNA (from six hiPSC lines of the training set, n = 6 for each line) were labelled using the FlashTag Biotin HSR RNA labelling kit (Affymetrix, Sunnyvale, CA, USA), according to the manufacturer’s instructions. Labelled RNA was processed for microarray hybridisation to miRNA 3.0 array (Affymetrix). An Affymetrix GeneChip fluidics station was used to perform streptavidin/phycoerythrin staining. The hybridisation signals on the microarray were scanned using a GeneChip Scanner 3000 (Affymetrix), and normalisation was performed using the miRNA array RMA + DABG analysis and the Expression Console software (Affymetrix). The National Center for Biotechnology Information Gene Expression Omnibus (NCBI GEO) accession number for the miRNA array data is GSE117739.

### GeneChip profiling and biostatistical analysis

Undifferentiated hiPSCs were maintained on Matrigel (Corning)-coated dishes in mTeSR1 medium (StemCell Technologies). Total RNA was isolated from hiPSC lines using an RNeasy mini kit (Qiagen) and treated with DNase I according to the manufacturer’s instructions. RNA samples (from six hiPSCs lines of the training set, n = 6 for each line) were converted into biotinylated cRNA using GeneChip 3′ IVT PLUS reagent kit (Affymetrix).

Labelled RNA was processed for microarray hybridisation to Human Genome U133 Plus 2.0 GeneChips (Affymetrix). An Affymetrix GeneChip fluidics station was used to perform streptavidin/phycoerythrin staining. The hybridisation signals on the microarray were scanned using a GeneChip Scanner 3000 (Affymetrix) and analysed using Expression console software (Affymetrix). Normalisation was performed after global scaling, with the arrays scaled to a trimmed average intensity of 500 after excluding the 2% probe sets with the highest and lowest values. The hybridisation experiments were performed with six samples of each hiPSC line. The NCBI GEO accession number for the microarray data is GSE88963. To identify the probe sets related to cardiac differentiation of hiPSCs, paired sample t-tests were conducted to compare high and low differentiation groups based on PCA ranking. Statistical significance was defined as *p* < 0.01 as determined by the Student’s *t*-test with a fold change (FC) > 2.

### CAGE profiling

Undifferentiated hiPSCs were maintained on MEF feeder (ReproCell)-coated dishes in primate ES medium (ReproCell). Eleven hiPSC lines of a training set, including four hiPSCs with different passages for 253G1 and two for 201B7 and R-12A were used for total RNA extraction and purification using the TRIzol tissue kit (Invitrogen) according to the manufacturer’s protocol. RNA quality was assessed using a NanoDrop spectrophotometer and the Agilent total RNA nano kit. The standard CAGE protocol was adapted for sequencing on an Illumina platform^49,65^, the details of which are provided in the Supplemental Experimental procedures (CAGE profiling and data processing).

### Data analysis

Data are expressed as mean ± standard error of mean (SEM). Statistical significance was determined using a two-tailed Student’s *t* test or analysis of variance (ANOVA), as appropriate. Differences between groups were considered statistically significant at *p*-values < 0.05.

## Supplementary information

Supplementary information

Supplementary video 1

Supplementary Dataset 1

## Data Availability

The authors confirm that the data supporting the findings of this study are available within the article and its Supplementary Materials.
