# Supplementary material for: CXCL4/PF4 is a predictive biomarker of cardiac differentiation potential of human induced pluripotent stem cells
Source: Sci Rep. 2019 Mar 15;9:4638. doi: 10.1038/s41598-019-40915-w (PMC6420577; doi:10.1038/s41598-019-40915-w)
Supplement: Supplementary file 1 — Supplementary information [file 41598_2019_40915_MOESM1_ESM.pdf]

CXCL4/PF4 is a predictive biomarker of cardiac differentiation potential of human induced pluripotent stem cells

Fumiya Ohashi[1,2,3,4], Shigeru Miyagawa[2], Satoshi Yasuda[1], Takumi Miura[1], Takuya Kuroda[1], Masayoshi Itoh[5], Hideya Kawaji[5,6], Emiko Ito[2], Shohei Yoshida[2], Atsuhiro Saito[2], Tadashi Sameshima[4], Jun Kawai[5], Yoshiki Sawa[2], and Yoji Sato[1,3,7,8,9*]

[1]Division of Cell-Based Therapeutic Products, National Institute of Health Sciences, 3-25-26 Tonomachi, Kawasaki-ku, Kawasaki, Kanagawa 210-9501, Japan

[2]Department of Cardiovascular Surgery, Osaka University Graduate School of Medicine, 2-2 Yamada-oka, Suita, Osaka 565-0871, Japan

[3]Department of Cellular & Gene Therapy Products, Osaka University Graduate School of Pharmaceutical Sciences, 1-6 Yamadaoka, Suita, Osaka 565-0871, Japan

[4]Terumo Corporation, 1500 Inokuchi, Nakai-machi, Ashigarakami-gun, Kanagawa 259-0151, Japan

[5]Preventive Medicine and Diagnosis Innovation Program, RIKEN Center, 1-7-22, Suehirocho, Tsurumi-ku, Yokohama, Kanagawa 230-0045, Japan

[6]Preventive Medicine and Applied Genomics Unit, RIKEN Center for Integrative Medical Sciences, 1-7-22 Suehirocho, Tsurumi-ku, Yokohama, Kanagawa 230-0045, Japan

[7]Department of Quality Assurance Science for Pharmaceuticals, Nagoya City University Graduate School of Pharmaceutical Sciences, 3-1 Tanabe-dori, Mizuho-ku, Nagoya, Aichi 467-8603, Japan

[8]Department of Translational Pharmaceutical Sciences, Graduate School of Pharmaceutical Sciences, Kyushu University, 3-1-1 Maidashi, Higashi-ku, Fukuoka, Fukuoka 812-8582, Japan

[9]LiSE Laboratory, Kanagawa Institute of Industrial Science and Technology, 3-25-13 Tonomachi, Kawasaki-ku, Kawasaki, Kanagawa 210-0821, Japan

[*]Corresponding Author: Yoji Sato, Ph.D, Division of Cell-Based Therapeutic Products, National Institute of Health Sciences, 3-25-26 Tonomachi, Kawasaki-ku, Kawasaki 210-9501, Japan. Phone: +81-44-270-6525. Fax: +81-44-270-6526. E-mail: yoji@nihs.go.jp

Supplementary Information

Cell culture and differentiation of hiPSCs. Undifferentiated hiPSCs in the training set were cultured on mouse embryo fibroblast (MEF) feeders (ReproCell, Tokyo, Japan) treated with mitomycin C in primate embryonic stem (ES) medium (ReproCell) supplemented with 5 ng/mL basic fibroblast growth factor (bFGF; ReproCell). The hiPSC lines were passaged twice a week on MEF feeders. Feeder-free hiPSCs in the test set were cultured on iMatrix-511 (Nippi, Inc., Tokyo, Japan) using StemFit (Ajinomoto Co., Inc., Tokyo, Japan).

When the hiPSCs were differentiated into cardiomyocytes, the MEF feeders were removed using human ES/iPS dissociation solution (ReproCell). Cell colonies were then dissociated into single cells using Accumax (Innovation Cell Technologies, San Diego, CA, USA). Feeder-free hiPSCs were dissociated into single cells using Accutase (Innovation Cell Technologies). Embryoid bodies (EBs) were generated in EZSPHERE (AGC Techno Glass, Shizuoka, Japan) with StemPro-34 medium (Gibco, Grand Island, NY, USA) containing 50 μg/mL ascorbic acid (Wako, Osaka, Japan), 2 mM L-glutamine (Gibco), and 400 μM L-thioglycerol (Sigma-Aldrich, Saint Louis, MO, USA) with 0.5 ng/mL bone morphogenic protein 4 (BMP-4; R&D Systems) and

10 μM Y-27632 (Wako).

The next day, 5 mL StemPro-34 medium containing 10 ng/mL BMP-4 (R&D Systems), 6 ng/mL bFGF, and 6 ng/mL or 12 ng/mL Activin A (R & D Systems) was added into the EZSPHERE. On day 4, the EBs were transferred to a low-attachment plate (Corning, NY, USA), and the medium was changed to StemPro-34 medium containing 4 μM IWR-1 (Sigma-Aldrich). After day 6, the medium was changed to StemPro-34 medium containing vascular endothelial growth factor (VEGF; R & D Systems; 5 ng/mL) and bFGF (10 ng/mL) after every 2 days. The EBs were differentiated under hypoxic conditions (5% O_2 and 5% CO_2) at 37°C in a hypoxic incubator (Thermo Fisher Scientific, Waltham, MA, USA). The differentiated EBs were collected on days 14–18.

To study the effect of cardiac differentiation, hiPSCs were preincubated in a medium containing 1% dimethyl sulphoxide (DMSO; Wako), 10 μM IWR-1, 5 μM IWP-2 (Sigma-Aldrich), 6 μM CHIR99021 (StemCell Technologies, Vancouver, Canada) or 10 μM Mitoblock-6 (Focus Biomolecules, Plymouth Meeting, PA, USA) for overnight, or in a medium containing 1 μM Recombinant Human PF4 (Peprotech, New Jersey, USA) or 1% DMSO for 2 days. Following preincubation, the hiPSCs were

differentiated into cardiomyocytes as described above.

Flow cytometry. After differentiation into cardiomyocytes, the EBs were dissociated with TrypLE Select (Gibco) for 5–10 min and neutralised with DMEM (Nacalai Tesque, Kyoto, Japan) containing 10% foetal bovine serum (FBS; Sigma-Aldrich). Thereafter, the cells were fixed with CytoFix fixation buffer (Becton Dickinson, East Rutherford, NJ, USA) for 30 min at 4°C. Then, the fixed cells were stained with the cardiac troponin T (cTnt) antibody CT3 (Santa Cruz Biotechnology, Inc., Santa Cruz, CA, USA) or normal mouse IgG (Santa Cruz Biotechnology, Inc.) in perm/wash buffer (Becton Dickinson). Alexa Fluor 488-conjugated goat anti-mouse IgG (Thermo Fisher Scientific) was used as a secondary antibody. The stained cells were analysed using a flow cytometer (Becton Dickinson). Data were analysed using a FACS Canto II system (Becton Dickinson).

Beating analysis. Differentiated EBs were monitored by a motion analysis system (SI8000 View; Sony, Tokyo, Japan). The proportion of beating EBs and the cross-sectional area and diameter of EBs in the video images were calculated, using SI8000C

analyzer (Sony) and a fluorescence microscope (BZX-710; Keyence, Osaka, Japan).

Immunofluorescence staining. For immunofluorescence staining, cells were fixed with 4% paraformaldehyde for 10 min at room temperature and washed with phosphate-buffered saline (PBS). After incubation with blocking solution containing 10% normal bovine serum albumin (Sigma-Aldrich) for 1 h at room temperature, the cells were permeabilised by incubation with 0.25% Triton X-100 for 10 min at room temperature. The cells were then incubated overnight with primary antibodies for cTnt (Santa Cruz Biotechnology), Nkx2.5 (Abcam, Cambridge, United Kingdom), alpha-smooth muscle actin (αSMA, Abcam), and vimentin (Abcam) at 4°C. Anti-rabbit or anti-mouse IgG secondary antibodies conjugated with fluorescein such as Alexa Fluor 488 or Alexa Fluor 555 (Thermo Fisher Scientific) were used for visualisation of the antigens of interest. Nuclei were counterstained with Hoechst 33258 (Dojindo, Kumamoto, Japan). Immunofluorescent images were examined under a confocal microscope (FV1200 or SD-OSR, Olympus, Tokyo, Japan).

Quantitative reverse transcription-polymerase chain reaction (qRT-PCR) analysis. Undifferentiated hiPSCs were lysed using QIAzol lysis reagent (Qiagen, Hilden,

Germany). RNA was extracted using a miRNeasy mini kit (Qiagen) according to the manufacturer's protocol. cDNA was synthesised using a SuperScript VILO cDNA synthesis kit (Thermo Fisher Scientific). qPCR was performed using a ViiA 7 real-time PCR system (Applied Biosystems, Carlsbad, CA, USA), PCR primers, SYBR Green PCR master mix (Applied Biosystems) or Taqman probe, and Taqman Gene Expression master mix (Applied Biosystems) as described in Supplementary Table 5 and Supplementary Table 6. Relative expression analysis was performed using the expression level of *GAPDH*, a housekeeping gene, as a reference.

Heat map and principal component analysis (PCA). A heat map was generated based on the standardised data using Excel 2016 (Microsoft, Redmond, WA, USA), representing the cardiac marker genes *TNT2*, *NKX2.5*, *GATA4*, *MYL2*, *MYH6*, and *MYH7*, and cardiac maturation marker genes *SCN5A*, *RYR2*, *PPARGC1*, *KCNJ2*, *HCN4*, *CACNA1C*, and *ATP2A2* expressed in the differentiated hiPSC lines. PCA was also performed, and the first principal component scores were calculated for the cardiac marker genes on days 9 and 17 for six hiPSC lines from the training set using the JMP 12 software (SAS Institute, Cary, NC, USA).

Pathway analyses. We determined the intracellular location and biological function of a group of genes with significantly changed expression using Qiagen's Ingenuity Pathway Analysis (IPA) software. Enriched human homologues were classified into functional categories based on either biological function or canonical pathways. A right-tailed Fisher's exact test was used to calculate *p*-values to determine the probability of each enriched human homologue associated with the dataset based on chance alone. We selected human homologues that had been functionally annotated as "differentiation" by IPA and further confirmed their roles based on functional descriptions in the NCBI and GeneCard databases.

CAGE profiling and data processing. cDNA was synthesised from total RNA using random primers in the presence of trehalose and sorbitol under high temperature. The ribose diols in the cap structure and the 3' end of the RNA were oxidised and then biotinylated by reacting the generated aldehyde groups with biotin hydrazide (Vector Laboratories, Burlingame, CA, USA). Single-stranded RNAs were digested with RNase ONE ribonuclease (Promega, Fitchburg, WI, USA). The biotinylated RNA/cDNA was selected using Dynabeads M-270 streptavidin (Thermo Fisher Scientific). After

capturing on the beads, the cDNA was released into the supernatant by heat denaturation. The supernatant was subjected to RNase ONE/H digestion, followed by purification using AMPure XP (Bio Rad, Hercules, CA, USA) and adaptor ligation to both ends of the cDNA. A double-stranded cDNA library was created using DeepVent (exo-) DNA polymerase. The CAGE cDNA libraries were sequenced using an Illumina HiSeq 2500 sequencer (Illumina). The data have been submitted to DDBJ Read Archive (DRA) under accession number DRA007185. The obtained reads were aligned with the reference human genome (GRCh37) using Burrows-Wheeler Alignment tool (BWA)[1], and low quality alignments with a mapping quality of 20 or less were discarded. The 5′-ends of the remaining alignments were counted based on the robust set of CAGE peaks defined in a previous study[2] and submitted to the Functional Annotation of Mammalian Genome 5 (FANTOM5) web resource[3]. The read counts were normalised as counts per million based on the normalisation (size) factor calculated using the RLE (relative log ratio) method[4]. Expression analysis was conducted using edgeR[5].

References

1. Li, H. & Durbin, R. Fast and accurate short read alignment with burrows–wheeler transform. *Bioinformatics* **25**, 1754–1760, https://doi.org/10.1093/bioinformatics/btp324 (2009).

2. Forrest, A.R., *et al*. A promoter-level mammalian expression atlas. *Nature* **507**, 462, https://doi.org/10.1038/nature13182 (2014).

3. Lizio, M., *et al*. Gateways to the FANTOM5 promoter level mammalian expression atlas. *Genome Biol.* **16**, 22, https://doi.org/10.1186/s13059-014-0560-6 (2015).

4. Anders, S. & Huber, W. Differential expression analysis for sequence count data. *Genome Biol.* **11**, R106, https://doi.org/10.1186/gb-2010-11-10-r106 (2010).

5. Robinson, M.D., McCarthy, D.J. & Smyth, G.K. edgeR: a Bioconductor package for differential expression analysis of digital gene expression data. *Bioinformatics* **26**, 139–140, https://doi.org/10.1093/bioinformatics/btp616 (2010).

Supplementary Figures, Tables and Video

Supplementary Table 1 Information about the hiPSC lines of the training set.

Cell Line	Vectors	Reprogramming factors	Cell source	Donor Racial	Donor Age	Donor Sex	Culture Method	Differentiaton Group	Reference
201B7	Retroviral	Oct3/4, Sox2, Klf4, c-Myc	Human dermal fibroblast	Caucasian	36	Female	MEF	High	Takahashi K, et al. Cell. 2007 Nov 30;131(5):861-72.
253G1	Retroviral	Oct3/4, Sox2, Klf4	Human dermal fibroblast	Caucasian	36	Female	MEF	High	Nakagawa M, et al. Nat Biotechnol. 2008 Jan;26(1):101-6. Epub 2007 Nov 30.
409B2	Episomal	Oct3/4, Sox2, Klf4, L-Myc, Lin28+ shRNA for p53	Human dermal fibroblast	Caucasian	36	Female	MEF	High	Okita K, et al. Nat Methods. 2011 May;8(5):409-12.
RIKEN-1A (R-1A)	Retroviral	Oct3/4, Sox2, Klf4, c-Myc	Umbilical cord blood-derived fibroblasts	Japanese	-	Female	MEF	Low	Fujioka T, et al. Hum Cell. 2010 Aug;23(3):113-8
RIKEN-2A (R-2A)	Retroviral	Oct3/4, Sox2, Klf4, c-Myc	Umbilical cord blood-derived fibroblasts	Japanese	-	Male	MEF	Low	Fujioka T, et al. Hum Cell. 2010 Aug;23(3):113-8
RIKEN-12A (R-12A)	Retroviral	Oct3/4, Sox2, Klf4, c-Myc	Umbilical cord blood-derived fibroblasts	Japanese	-	Male	MEF	Low	Fujioka T, et al. Hum Cell. 2010 Aug;23(3):113-8

Supplementary Table 2 Spearman's rank correlation analysis between cardiac differentiation capacity at 17 d post-differentiation and the expression of EB-related genes at 4 d post-differentiation.

Lineage	Marker	rs	*p*-value
Mesoderm	*FLK1*	0.75	0.05
	PDGFR-A	0.29	0.53
	GOOSECOID	0.21	0.64
	BRACHYURY	0.11	0.82
Endoderm	*SOX17*	0.39	0.38
	AMN	-0.89	0.01
	SOX7	-0.11	0.82
	HNF3	-0.36	0.43
Ectoderm	*SOX1*	-0.29	0.53
	PAX6	-0.79	0.04
	ZIC1	-0.57	0.18

Spearman correlation coefficient (rs) and *p*-value were calculated for statistical analysis.

Supplementary Table 3 Differentially expressed miRNAs and snoRNAs in the high and low purity groups.

Probe Set ID	Fold Change	Type	Subcellular Location	Role in cell
ACA24	↓−2.0	small nucleolar RNA	-	-
hsa-miR-629-star	↓−2.1	mature microRNA	Cytoplasm	-
mml-miR-204	↓−2.6	mature microRNA	Cytoplasm	expression in, differentiation, antiapoptosis, activation in, proliferation, maturation
ACA61	↓−4.0	small nucleolar RNA	-	-
hsa-miR-139	↓−11.4	mature microRNA	Cytoplasm	growth

Three miRNAs and 2 snoRNAs showed significant difference in their levels in the high and low purity groups (p < 0.05, FC > 2).

Supplementary Table 4 Information about the hiPSC lines of the test set.

Six hiPSC lines of the test set:

Cell Line	Vectors	Reprogramming factors	Cell source	Donor Racial	Donor Age	Donor Sex	Culture Method	Differentiaton Group	Reference
454 E2	Episomal	Oct3/4, Sox2, Klf4, L-Myc, Lin28+ shRNA for p53	Dental pulp cell	Japanese	16	Female	MEF	Low	Kajiwara M, et al. Proc. Natl. Acad. Sci. USA. 2012 Jul 31;109(31):12538-43.
606A1	Episomal	Oct3/4, Sox2, Klf4, L-Myc, Lin28	CD34+ cord blood cells	Japanese	-	Female	MEF	Low	Kajiwara M, et al. Proc. Natl. Acad. Sci. USA. 2012 Jul 31;109(31):12538-43.
648A1	Episomal	Oct3/4, Sox2, Klf4, L-Myc, Lin28+ shRNA for p53	Peripheral blood mononuclear cell	Japanese	30s	Male	MEF	Low	Okita K, et al. Stem Cells. 2013 Mar;31(3):458-66.
Tic	Retroviral	Oct3/4, Sox2, Klf4, c-Myc	human foetus lung cells (MRC-5)	-	14 weeks foetus	Male	MEF	Low	Fujioka T, et al. Hum Cell. 2010 Aug;23(3):113-8
ChiPSC18	Retroviral	Oct4, Sox2, Klf4, Myc	Human dermal fibroblast	North African	32	Male	iMatrix511	High	Asplund A, et al. Stem Cell Rev. 2016 Feb;12(1):90-104.
ChiPSC21	Retroviral	Oct4, Sox2, Klf4, Myc	Human dermal fibroblast	North African	26	Male	iMatrix511	Low	Asplund A, et al. Stem Cell Rev. 2016 Feb;12(1):90-104.
ChiPSC22	Retroviral	Oct4, Sox2, Klf4, Myc	Human dermal fibroblast	North African	32	Male	iMatrix511	Low	Asplund A, et al. Stem Cell Rev. 2016 Feb;12(1):90-104.

Supplementary Table 4 Information about the hiPSC lines of the test set (continuted).

Seven hiPSC lines of the test set:

Cell Line	Vectors	Reprogramming factors	Cell source	Donor Racial	Donor Age	Donor Sex	Culture Method	Differentiaton Group	Reference
PCi-1432	Episomal	Oct4, Sox2, Klf, cMyc	Peripheral blood mononuclear cell	South European	30-50	Male	iMatrix511	Low	-
PCi-1503	Episomal	Oct4, Sox2, Lin28, Klf4, L-Myc	Peripheral blood mononuclear cell	Asian	-	Female	iMatrix511	Low	-
PCi-1533	Episomal	Oct4, Sox2, Lin28, Klf4, L-Myc	Peripheral blood mononuclear cell	African	26	Female	iMatrix511	Low	-
ATCC-HYR0103	Retroviral	Oct4, Sox2, Klf4, Myc	Primary hepatic Fibroblast	Hispanic	31	Male	MEF	Low	-
Ff-201B7	Retroviral	Oct3/4, Sox2, Klf4, c-Myc	Human dermal fibroblast	Caucasian	36	Female	iMatrix511	High	Takahashi K, et al. Cell. 2007 Nov 30;131(5):861-72.
Ff-253G1	Retroviral	Oct3/4, Sox2, Klf4	Human dermal fibroblast	Caucasian	36	Female	iMatrix511	High	Nakagawa M, et al. Nat Biotechnol. 2008 Jan;26(1):101-6. Epub 2007 Nov 30.

Supplementary Table 5 List of oligonucleotide primers used in the current study.

Gene	Forward primer sequences (5′→3′)	Reverse primer sequences (5′→3′)
NKX2.5	ACCTCAACAGCTCCCTGACTC	ATAATCGCCGCCACAAACTCTCC
TNNT2	TTCACCAAAGATCTGCTCCTCGCT	TTATTACTGGTGTGGAGTGGGTGTGG
MYL2	TGTCCCTACCTTGTCTGTTAGCCA	ATTGGAACATGGCCTCTGGATGGA
MYH6	TCAGCTGGAGGCCAAAAGTAAAGGA	TTCTTGAGCTCTGAGCACTCGTCT
MYH7	TCGTGCCTGATGACAAACAGGAGT	ATACTCGGTCTCGGCAGTGACTTT
GATA4	AGGCCTCTTGCAATGCGGA	CTGGTGGTGGCGTTGCTGG
LIN28	CACGGTGCGGGCATCTG	CCTTCCATGTGCAGCTTACTC
GAPDH	CAATGACCCCTTCATTGACC	TTGATTTTGGAGGGATCTCG
NANOG	AAAGAATCTTCACCTATGCC	GAAGGAAGAGGAGAGACAGT
SOX1	CACAACTCGGAGATCAGCAA	GGTACTTGTAATCCGGGTGC
PAX6	GTCCATCTTTGCTTGGGAAA	TAGCCAGGTTGCGAAGAACT
ZIC1	CTGGCTGTGGCAAGGTCTTC	CAGCCCTCAAACTCGCACTT
FLK1	TGATCGGAAATGACACTGGA	CACGACTCCATGTTGGTCAC
PDGFR-A	ACAGGTTGGTGTGGGTTCAT	CTGCATCTTCCAAAGCATCA
BRACHYURY	AATTGGTCCAGCCTTGGAAT	CGTTGCTCACAGACCACA
GOOSECOID	GAGGAGAAAGTGGAGGTCTG	CTCTGATGAGGACCGCTTCTG
SOX17	CTCTGCCTCCTCCACGAA	CAGAATCCAGACCTGCACAA
HNF3	GGAGCGGTGAAGATGGAA	TACGTGTTCATGCCGTTCAT
AMN	GACTCTGACCGCTTCTCCTG	CACTAGGCGGAAAGAAGACG
SOX7	ACGCCGAGCTCAGCAAGAT	TCCACGTACGGCCTCTTCTG
OCT-4	CAATTTGCCAAGCTCCTGAAG	AAAGCGGCAGATGGTCGTT

Supplementary Table 6 List of Taqman primers used in the current study.

Gene	Assay reference
ANKRD1	Hs00173317_m1
ATP2A2	Hs00544877_m1
BCOR	Hs00372378_m1
C7orf50	Hs00260594_m1
CACNA1C	Hs00167681_m1
CHCHD2	Hs00853326_g1
FGF17	Hs00182599_m1
FOXQ1	Hs00536425_s1
GAPDH	Hs02758991_g1
GATA6	Hs00232018_m1
GLIPR1	Hs01564146_g1
HCN4	Hs00975492_m1
IGFBP5	Hs00181213_m1
KCNJ2	Hs01876357_s1
KDM6A	Hs00253500_m1
MYL4	Hs04187281_m1
PF4	Hs00427220_g1
PLCB1	Hs01001930_m1
POMZP3	Hs02341150_m1
PPARGC1	Hs01016719_m1
PTGR1	Hs00400932_m1
RBMX	Hs00953944_g1
RC3H1	Hs01367694_m1
RIPK1	Hs01041869_m1
RYR2	Hs00181461_m1
SCN5A	Hs00165693_m1
SKIL	Hs01045418_m1
TMEM64	Hs01595139_m1
WNT3	Hs00902257_m1
ZNF229	Hs00970996_g1

Supplementary Video 1 Beating EBs derived from hiPSCs treated with PF4 (right) or DMSO as a control (left) for 2 days prior to cardiac differentiation induction.

Supplementary Figure 1

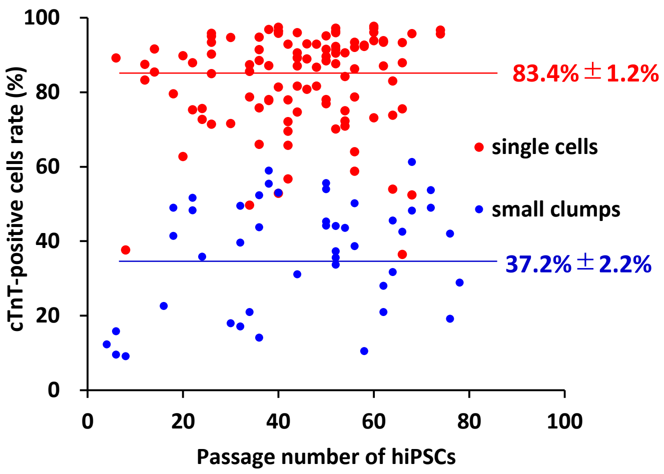

Supplementary Figure 1 Effect of passage number and differentiation protocols on cardiac differentiation efficiency. Quantification of cTnT expression measured using flow cytometry in 253G1 hiPSCs prepared using two different protocols. Red dots: single cells (83.4 ± 1.2%, n = 110) ; blue dots: small clumps (37.2 ± 2.2%, n = 46) . Data are expressed as mean ± SEM.

Supplementary Figure 2

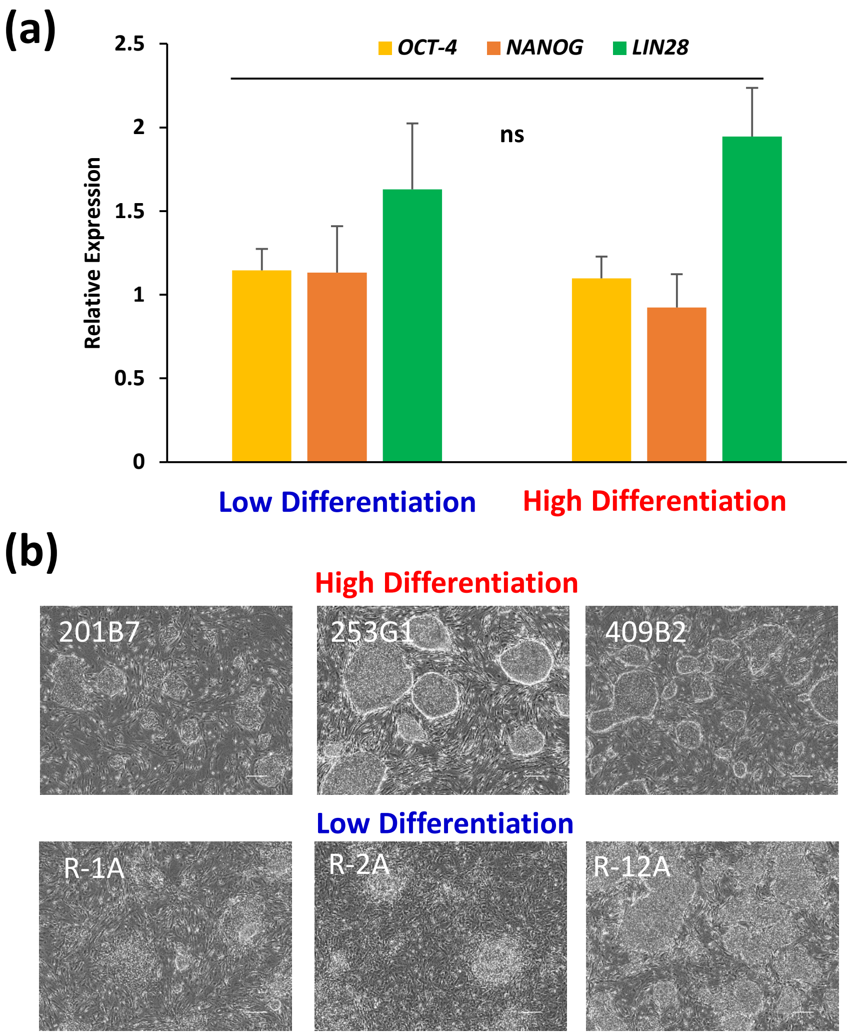

Supplementary Figure 2 Undifferentiated gene expression profiles in a training set of hiPSCs lines. (a) Comparison of undifferentiated related genes of hiPSCs lines (ns, not significant). Data are expressed as mean ± SEM (n = 3). All mRNA values are shown as fold change relative to the expression of R-2A in Low differentiation group. (b) Images of hiPSCs from experiments in the training set of hiPSC lines. Scale bars = 300 μm.

Supplementary Figure 3

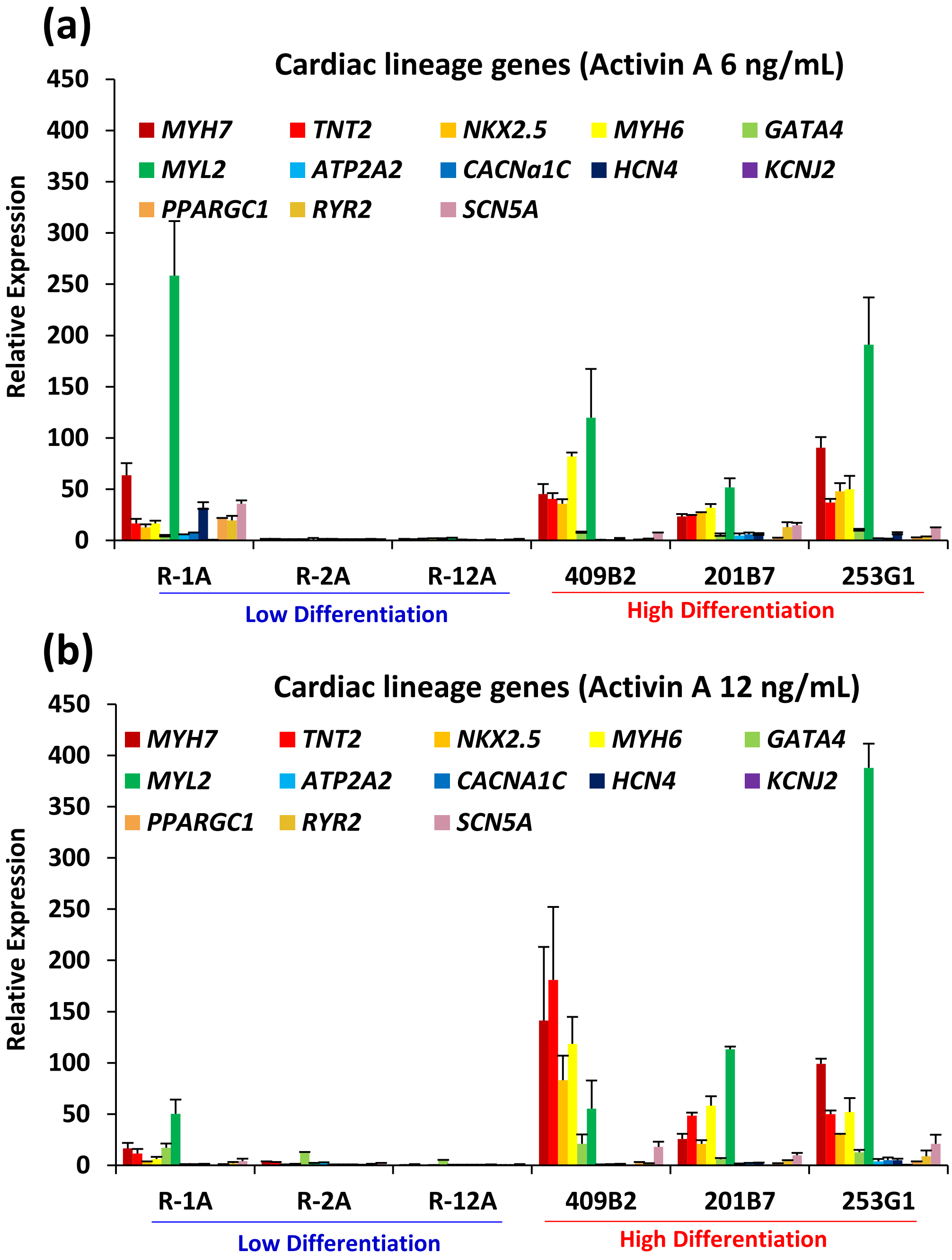

Supplementary Figure 3 Cardiac lineage gene expression in EBs at 17 d post-differentiation. Differences in cardiac lineage differentiation potential among hiPSC lines induced with different concentrations of Activin A; 6 ng/mL (a) and 12 ng/mL (b). All mRNA values are shown as fold change relative to the expression of R-2A at 6 ng/mL Activin A. Data are expressed as mean ± SEM (n = 3).

Supplementary Figure 4

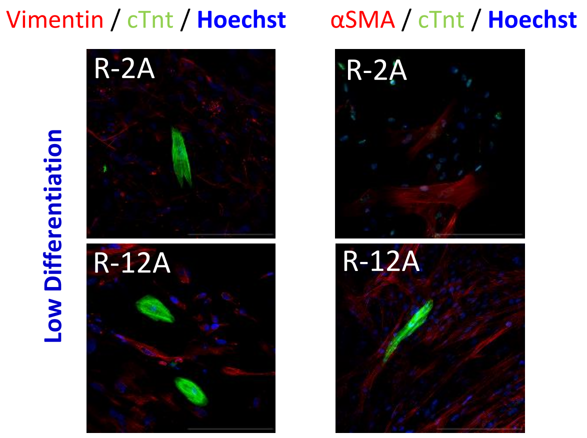

Supplementary Figure 4 Immunofluorescence of cardiomyocytes derived from low differentiation group (R-2A and R-12A) for cardiac-specific markers and smooth muscle cell and fibroblast markers. Left micrographs show cTnT (green), vimentin (red), and Hoechst (blue) staining. Right micrographs show cTnT (green), αSMA (red), and Hoechst (blue) staining. Scale bars = 100 μm.

Supplementary Figure 5

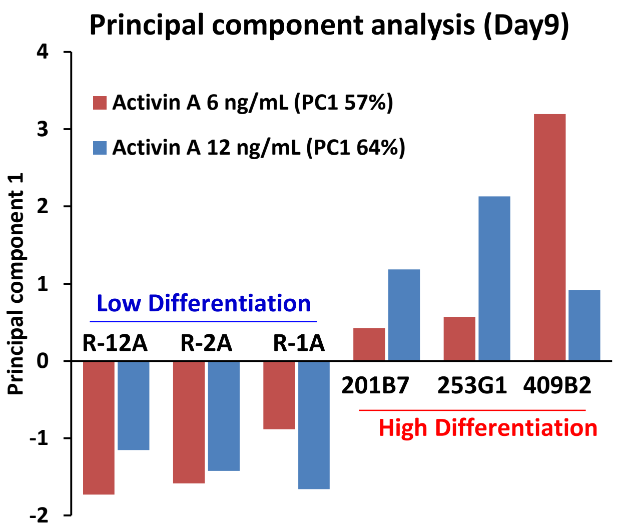

Supplementary Figure 5 Principal component analysis of the cardiac differentiation ability among six hiPSC lines at 9 d post-differentiation (FC1, first principal component scores).

Supplementary Figure 6

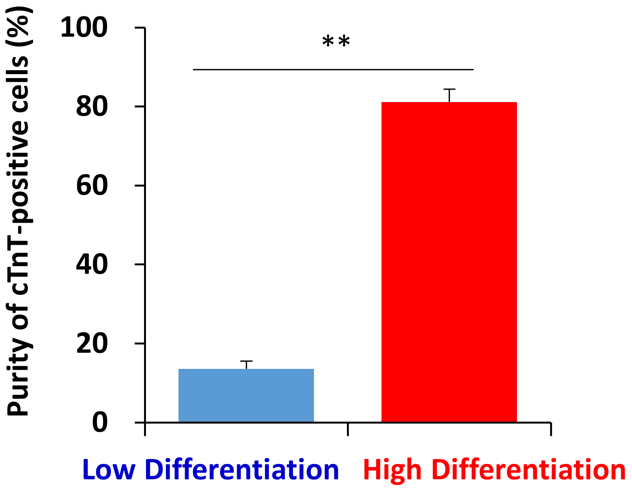

Supplementary Figure 6 Percentage of cTnT-positive cells generated from hiPSCs in high and low differentiation groups at 17 d post-differentiation in the training set of hiPSC lines. Data are expressed as mean ± SEM (n = 6). $**p < 0.01$, *t*-test.

Supplementary Figure 7

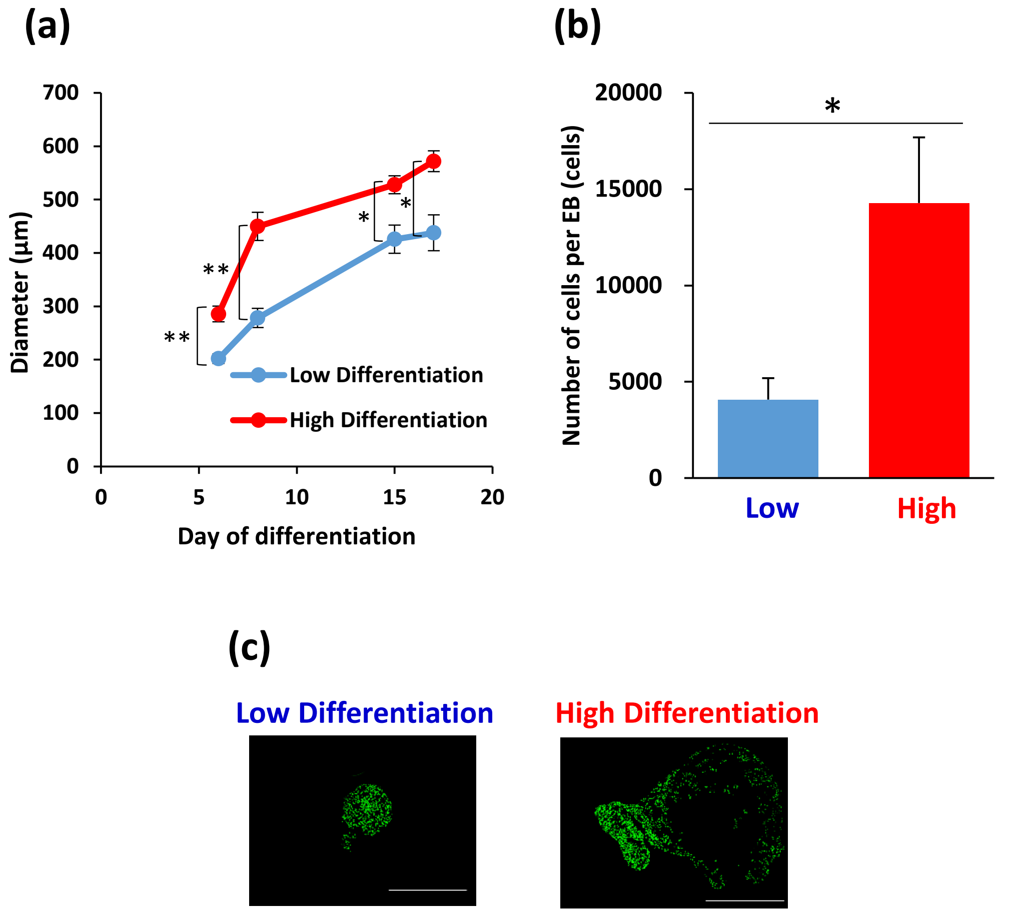

Supplementary Figure 7 (a) The diameter of EBs in the high and low differentiation groups during the cardiac differentiation. Data are expressed as mean ± SEM (n = 6). $^{**}p < 0.01$; $^{*}p < 0.05$, *t*-test. (b) Number of cells per EB at 17 d post-cardiac differentiation. Data are expressed as mean ± SEM (n = 6). $^{*}p < 0.05$, *t*-test. (c) The section from EBs at 17 d post-cardiac differentiation was immunostained with Hoechst (green). Scale bars = 500 µm.

Supplementary Figure 8

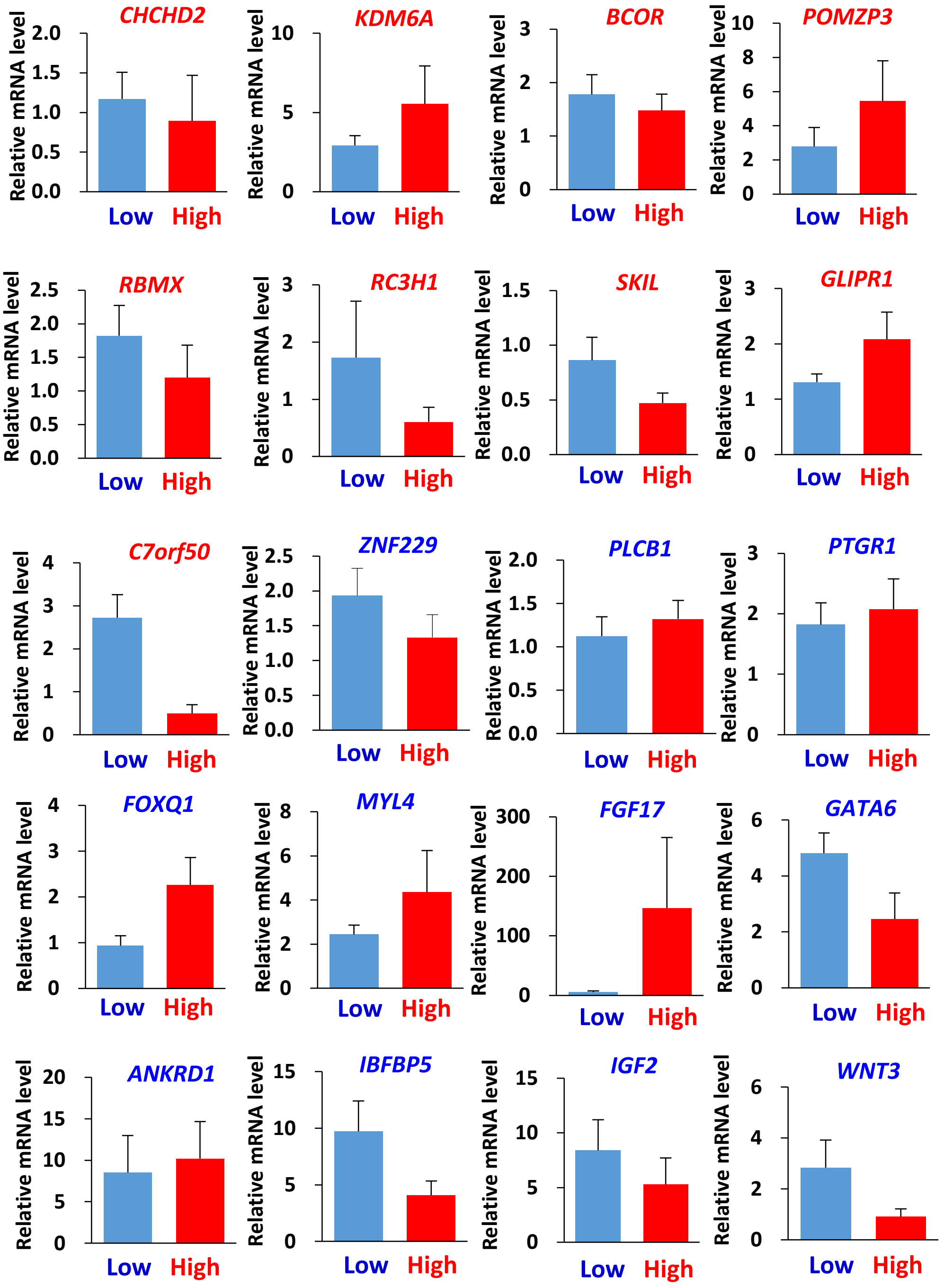

Supplementary Figure 8 Expression profile of 20 candidate genes based on qRT-PCR for positive predictors (red) and negative predictors (blue).

Supplementary Figure 9

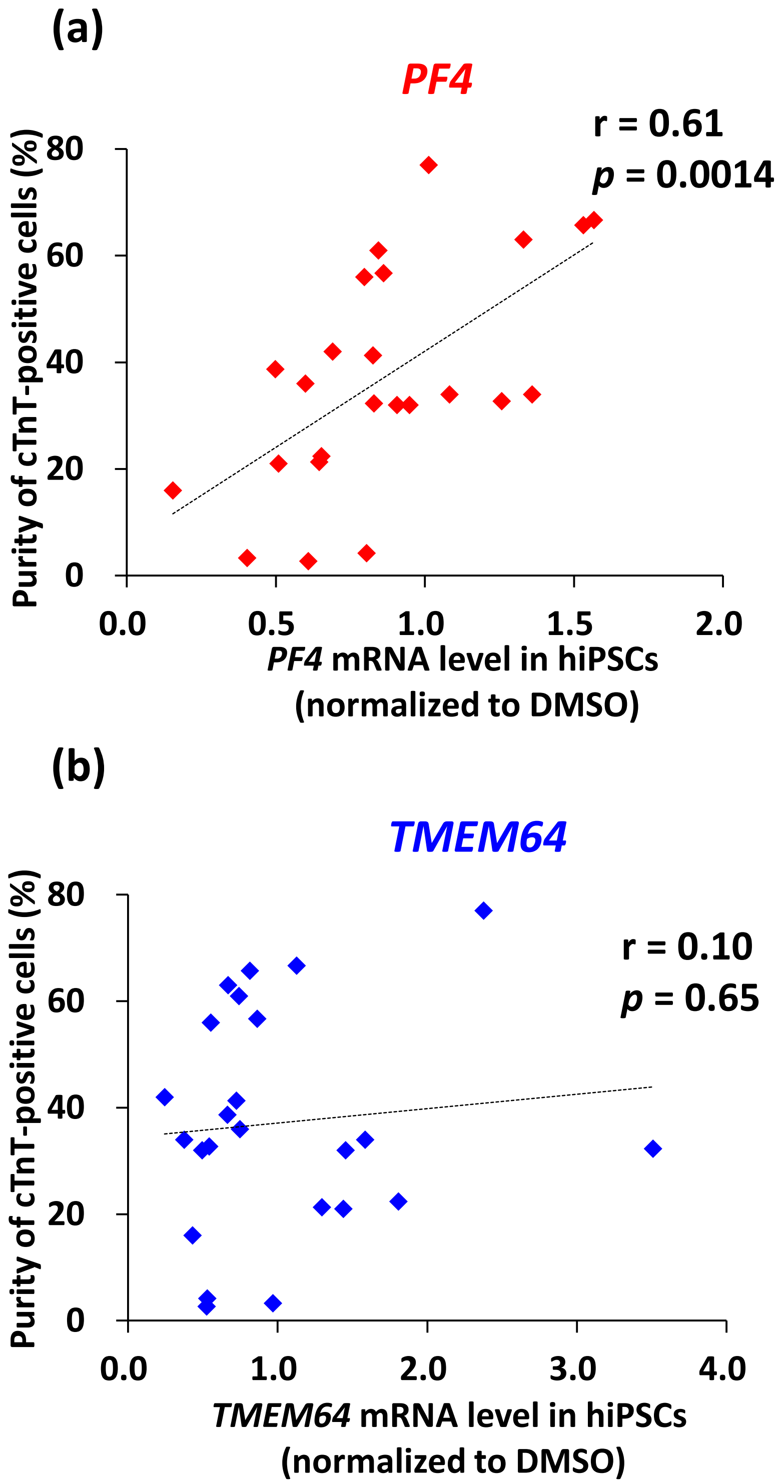

Supplementary Figure 9 Pearson correlation analysis between *PF4* (a) and *TMEM64* (b) mRNA levels in undifferentiated hiPSCs and their cardiac differentiation efficiency along with r and *p* values.

Supplementary Figure 10

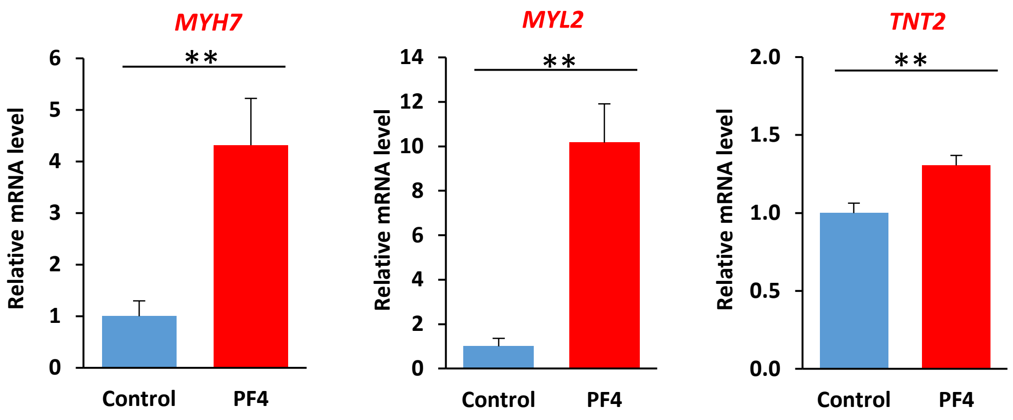

Supplementary Figure 10 Expression of cardiomyocyte-specific genes (*MYH7*, *MYL2* and *TNT2*) at 14 d post-differentiation in EBs derived from 253G1 hiPSCs treated with PF4 (1 μM) or DMSO (1 %) as a control for 2 days prior to differentiation induction. The mRNA values are shown as fold changes relative to the expression of the controls. Data are expressed as mean ± SEM (n = 9). ***p* < 0.01, *t*-test.

Supplementary Figure 11

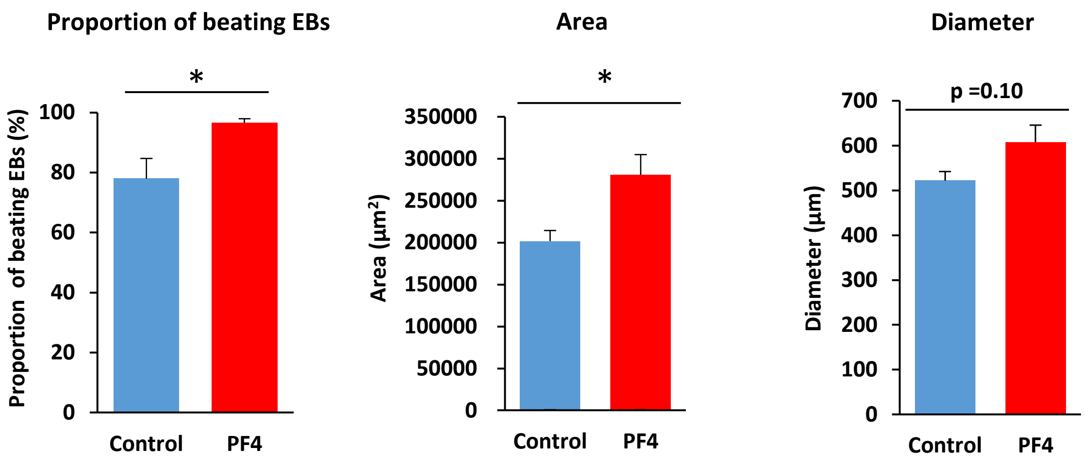

Supplementary Figure 11 Proportion of beating EBs, and cross-sectional area and diameter of EBs derived from 253G1 hiPSCs treated with PF4 (1 μM) or DMSO (1 %) as a control for 2 days prior to differentiation induction. Data were obtained from EBs at 14 d post-differentiation and are expressed as mean ± SEM (n = 6). *$p < 0.05$, *t*-test.
